# Continuous flow catalysis with CuBTC improves reaction time for synthesis of xanthene derivatives

**DOI:** 10.3389/fchem.2023.1259835

**Published:** 2023-10-16

**Authors:** Jonathan E. Thai, Madeline C. Roach, Melissa M. Reynolds

**Affiliations:** ^1^ Department of Chemistry, Colorado State University, Fort Collins, CO, United States; ^2^ School of Biomedical Engineering, Colorado State University, Fort Collins, CO, United States; ^3^ Dapartment of Chemical and Biological Engineering, Colorado State University, Fort Collins, CO, United States

**Keywords:** metal-organic frameworks, catalysis, continuous flow, flow catalysis, xanthene

## Abstract

The copper-based metal-organic framework (MOF) CuBTC (where H_3_BTC = benzene-1,3,5-tricarboxylate) has been shown to be an efficient heterogeneous catalyst for the generation of 1,8-dioxo-octa-hydro xanthene derivatives, which are valuable synthetic targets for the pharmaceutical industry. We have applied this catalytic capability of CuBTC to a continuous flow system to produce the open chain form of 3,3,6,6-tetramethyl-9-phenyl-3,4,5,6,7,9-hexahydro-1*H*-xanthene-1,8(2*H*)-dione, a xanthene derivative from benzaldehyde and dimedone. An acid work-up after producing the open chain form of the xanthene derivative was used to achieve ring closure and form the final xanthene product. The CuBTC used to catalyze the reaction under continuous flow was confirmed to be stable throughout this process via analysis by SEM, pXRD, and FT-IR spectroscopy, elemental analysis, and XPS. The reaction to produce the open-chain form of the xanthene derivative produced an average yield of 33% ± 14% under the continuous flow (compared to 33% ± 0.12% of performing it under batch conditions). Based on the data obtained from this work, the continuous flow system required 22.5x less time to produce the desired xanthene derivative at comparable yields to batch reaction conditions. These results would allow for the xanthene derivative to be produced much faster, at a lower cost, and require less personal time while also removing the need to perform catalyst remove post reaction.

## Introduction

**FIGURE 1:**
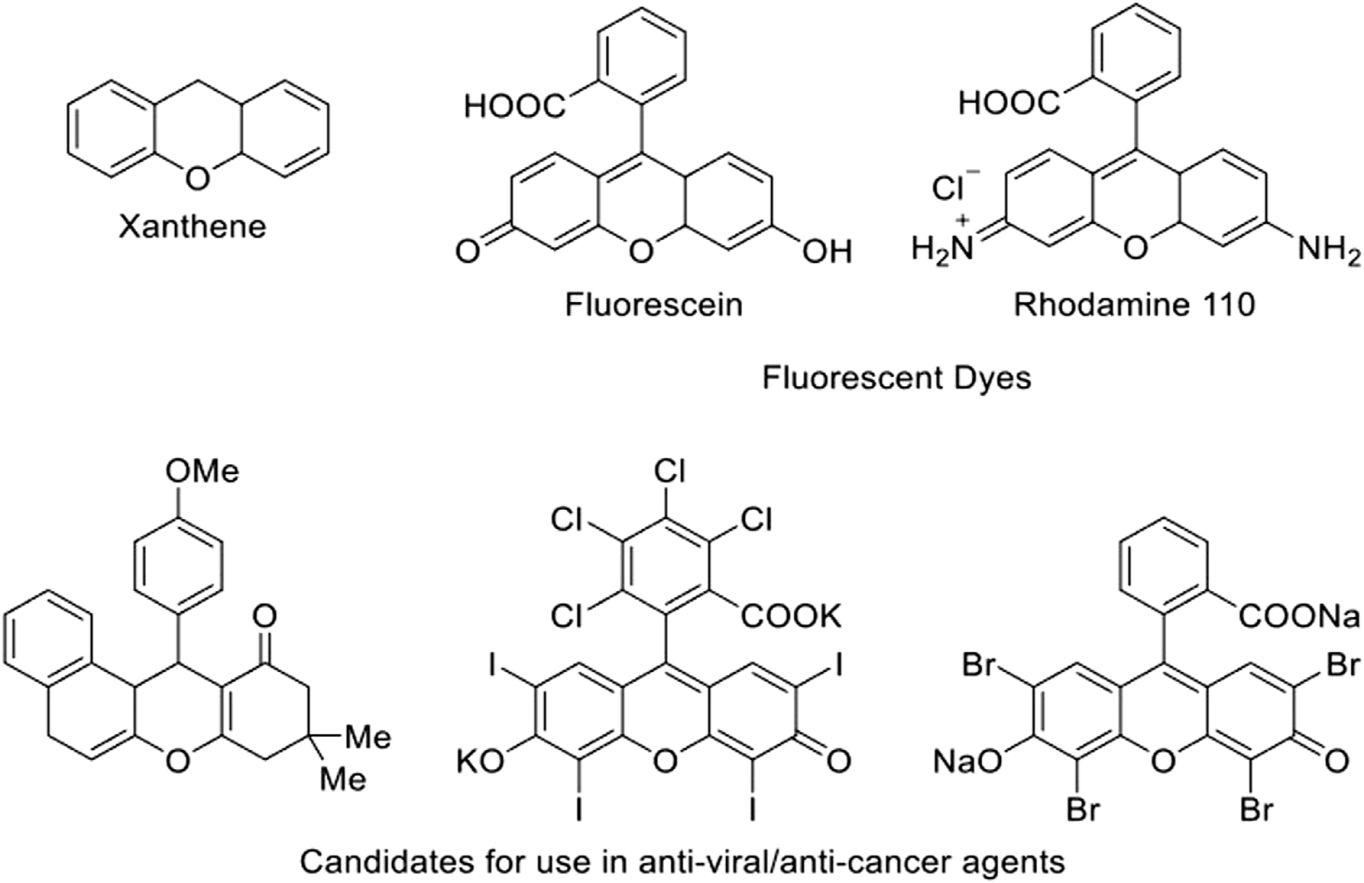
Chemical structure of xanthene and example structures of xanthene derivatives and their uses (Gunjegaonkar et al., 2018).

While originally used in dyes, interest in xanthene derivatives has grown in recent years due to their expanded known applications in materials and medicinal chemistry (Abdel-Galil et al., 1982; Barrett et al., 2000; Callan et al., 2005; Aksenov, 2014; Xu et al., 2016; Chen et al., 2019; Yang et al., 2019; Rahnamafar et al., 2020; Ghamari Kargar et al., 2021; Wei et al., 2022). Xanthene derivatives (Figure 1) have found uses in the fields of biodegradable agrochemicals, photodynamic therapy, solar cells, luminescent sensors, lasers, anti-inflammatory agents, antibacterial drugs, antinociceptive, antitumor, and anti-viral treatments. Due to their wide variety of applications, developing new routes to produce xanthene derivatives has become an active area of research in recent years (Ilangovan et al., 2011; Ghafuri et al., 2021).

**FIGURE 2:**
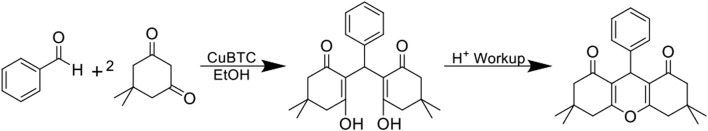
Reaction for the condensation between benzaldehyde and two equivalents of dimedone to produce the open chain form of 3,3,6,6-tetramethyl-9-phenyl-3,4,5,6,7,9-hexahydro-1*H*-xanthene-1,8 (2*H*)-dione followed by an acid work-up to close the ring.

Several routes for the synthesis of xanthene derivatives using heterogeneous catalysts have been published in the literature, but have shown to have limitations such as variable yields (0%–96%) (Yang et al., 2019; Ghamari Kargar et al., 2021; Wei et al., 2022), variable reaction times (10 min–24 h) (Venkatesan et al., 2008; Lee et al., 2020; Cao et al., 2021) and potentially harsh reaction conditions. (Ilangovan et al., 2011; Ghafuri et al., 2021). In 2020, Ghafuri *et al.* used CuBTC (where H_3_BTC = benzene-1,3,5-tricarboxylate, sometimes referred to as HKUST-1 or Basolite C 300) to catalyze condensations between benzaldehyde and dimedone to produce **2** (Figure 2). (Ghafuri et al., 2021) CuBTC is a commercially available metal-organic framework (MOF) that has been shown to be useful for Lewis acid catalysis. (Arnanz et al., 2012; Pérez-Mayoral et al., 2012; Položij et al., 2013; Liu et al., 2014; Dhakshinamoorthy et al., 2017; Zhu et al., 2017; Bavykina et al., 2020). The xanthene derivative **2** was identified by Merck & Co. to be a valuable target for use in the development of pharmaceuticals for antiviral and antibacterial drugs. (Ghafuri et al., 2021). When using CuBTC as the catalyst, the reaction to produce **2** was reported to have good yield (92%), a relatively short reaction time (15 min reflux), used a non-toxic solvent (ethanol), and mild reaction conditions (80°C) (Ghafuri et al., 2021). We hypothesized that transferring the CuBTC catalyzed condensation of xanthene derivatives into a continuous flow system would result in in further reduction in reaction times, as has been previously reported in other systems (Tan et al., 2019; Williams et al., 2019). Performing reactions under continuous flow conditions has increased in popularity, especially for pharmaceutical targets as continuous flow systems offer advantages compared to batch conditions (Dubois et al., 2023). As an example, continuous flow systems are commonly reported to offer reduced reaction times due to improved mixing and/or more efficient mass transfer relative to more traditional batch method conditions (Zhang et al., 2014; Dubois et al., 2023). Furthermore, as flow equipment becomes more advanced, reaction scalability has become more feasible (Gutmann et al., 2015).

Previous literature has leaned towards not using direct packing due to issues in very high column pressures (Ji et al., 2019). This work shows the potential to use direct packing of the MOF for catalytic flow systems as there was no measurable increase in pressure in the flow system when this reaction was performed. This provides precedence for the use of direct packing of MOFs in catalytic beds which allow for a larger amount of MOF to be used which can allow for the use of higher flow rate and thus, faster production times. Direct packing is also arguably a simpler method to pack a column, also allowing for these systems to be more easily produced than requiring alternative packing methods such as growing the MOFs onto silica.

**FIGURE 3:**
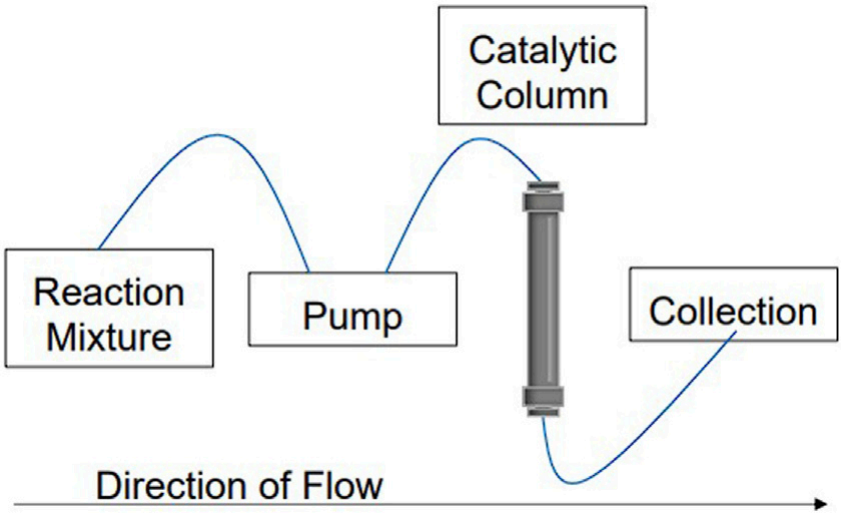
An example of the continuous flow system used with a catalytic column in place. For the setup used in this work, the pump was from an HPLC and the catalytic column was directly packed with CuBTC MOF.

Herein, we introduce a method to synthesize the xanthene derivative 3,3,6,6-tetramethyl-9-phenyl-3,4,5,6,7,9-hexahydro-1*H*-xanthene-1,8 (2*H*)-dione using a CuBTC catalyzed continuous flow system. An HPLC pump was used to simulate continuous flow conditions, pumping benzaldehyde and dimedone through a packed bed of CuBTC in a stainless steel HPLC column (Figure 3). The reaction mixture was injected in 3 mL increments to match the reaction mixture volume of the reaction under batch conditions and then characterized using TOF-MS. CuBTC was synthesized and characterized using pXRD, SEM, FT-IR, and XPS before and after being used in the column. Elemental analysis was also used to provide further evidence of the stability of the MOF after it was used by analyzing the eluent from the column. The synthesis was successful, and the time required to generate the desired product was decreased significantly as compared to batch conditions. Noteworthy, the continuous flow method also shows viability for medical or industrial applications, as there are no detectable metal impurities in the product even though a metal-based catalyst was used.

## Materials and methods

### Materials

All chemicals and solvents were purchased from commercial vendors and were then used without any further purification. *N,N*-dimethylformamide, >99.6%; dimedone, >98%; and ethanol, 100% was purchased form Fisher Scientific (Waltham, MA). 1,3,5-benzenetricarboxylic acid, >98%; and copper (II) nitrate trihydrate, >99%; 3,3,6,6-tetramethyl-9-phenyl-3,4,5,6,7,9-hexahydro-1*H*-xanthene-1,8 (2*H*)-dione; and benzaldehyde, >99% were purchased from Sigma-Aldrich (St. Louis, MA). All deionized water used was purified by a Millipore Milli-Q-IQ 7000 water purification system to 18.2 MΩ-cm resistivity prior to use.

### Synthesis of CuBTC MOF

The CuBTC MOF used in all experiments was synthesized using a method adapted from the literature. (Nguyen et al., 2012). In short, 1,3,5-benzenetricarboxylic acid (12.85 mmol, 0.62 equiv.) and copper (II) nitrate trihydrate (20.695 mmol, 1.0 equiv.) were suspended in a mixture of deionized water (23 mL) DMF (33 mL), and ethanol (36 mL) in a sealable Pyrex bottle. The mixture was sonicated for at least 1 h to fully disperse and suspend the reagents. The mixture was then placed into a pre-heated oven at 90°C for 24 h. After 24 h, the oven was turned off and the product was cooled to room temperature inside the oven naturally. Once cooled to room temperature, the solid product was then collected using a find glass filter frit. The solid product was then rinsed with DMF (3 × 50 mL). The solid product was then washed in ethanol using Soxhlet extraction for at least 24 h. The solid product was then collected using a fine glass filter frit. The solid was then collected and dried at 150°C under reduced pressure, producing a dark blue powder. The collected product was then characterized using SEM and pXRD.

### Using CuBTC to catalyze the condensation between dimedone and benzaldehyde under continuous flow conditions

CuBTC was activated at 150°C under reduced pressure overnight in a vacuum oven. The oven was then turned off and the CuBTC was allowed to cool down to room temperature. A stainless-steel HPLC column (I.D. x L, 4.6 × 50 mm) was then directly packed with CuBTC and connected to an HPLC pump via stainless steel tubing. The column was then pre-heated to 80°C while being rinsed with ethanol at 0.5 mL/min for at least 15 min prior to running the reaction. A reaction mixture was created by dissolving dimedone (20.0 mmol, 2 equiv.) and benzaldehyde (10.0 mmol, 1 equiv.) in ethanol (30 mL), yielding a clear reaction mixture in a Pyrex bottle. The reaction mixture was then pumped using an HPLC through the column using stainless steel tubing with I.D. 1 mm at a flow rate of 0.5 mL/min. Reaction mixture was pumped through the system for 6 min and then rinsed with ethanol for 30 min to ensure the entirety of the reaction was washed out of the column. Product was then characterized using NMR, IR, and TOF-MS. ^1^H NMR (CDCl_3_) δ 1.13 (s, 6H), 1.26 (s, 6H), 2.43 (m, 8H),5.57 (s, 1H), 7.18 (m, 2H), 7.29 (m, 3H).

### Characterization by attenuated total reflectance fourier transform-infrared spectroscopy (ATR-FTIR)

ATR-FTIR spectra were recorded within the range of 600–4,000 cm^−1^ using a ThermoFisher Scientific Nicolet 6,700 spectrometer. All samples were characterized on a diamond crystal and the aperture was set to 20. Spectra obtained were an average of 64 scans of the sample.

### Characterization by scanning electron microscopy (SEM)

SEM imaging was performed using a JEOL JSM-6500F microscope. An accelerating voltage of 15.0 kV was used to image the MOFs before and after use. All samples were prepared on a small piece of carbon tape which were then placed under vacuum and coated with 20 nm of gold prior to imaging. At least three representative images were taken with at least three different magnifications for each sample were taken.

### Characterization by powdered X-ray diffraction (pXRD)

A Bruker D8 Discover DaVinci Powder X-ray Diffractometer with Cu Kα radiation (λ = 1.5406 Å was used, operated at 40 kV and 40 mA. A 0.6-mm divergent slit was placed on the primary beam sided and a high-resolution energy dispersive LYNXEYE-XE-T detector was placed on the diffracted beam side during the XRD studies. Samples were seated on a B-doped silicon zero-diffraction plate and held in place with a small amount of vacuum grease. The 2θ was set between 4 and 50° and scanned at steps of 0.100°.

### Characterization by time of flight-mass spectrometry (TOF-MS)

An Agilent^®^ 6,224 time-of-flight mass spectrometer equipped with a dual ESI source operating in positive ionization mode was used for all TOF-MS analysis. The detection range for the scans was set for 50–1,000 *m/z*. The nebulizer gas temperature was set to 300°C, drying gas flow was set to 10 L/min, the nebulizer pressure was set to 45 psi, and the capillary voltage was set to 4500 V. All samples were analyzed under these conditions.

### Characterization by elemental analysis

Elemental analysis was performed by Huffman Hazen Laboratories in Golden, CO. Starting materials (benzaldehyde and dimedone) and reaction solvent (ethanol) were analyzed as controls. These samples and the collected eluent were then analyzed to determine the amount of free copper present in the samples.

### Characterization by X-ray photoelectron spectroscopy (XPS)

Surface elemental compositions were measured by a PE-5800 x-ray photoelectron spectrometer. XPS was performed in the Colorado State University Analytical Resources Core (ARC, RRID: SCR_201758) by Dr. Rebecca Miller. CuBTC MOF was analyzed from before and after it was used as a catalysis in the continuous flow system. High resolution scans of Carbon 1 s and Copper 2p regions. Samples were dried under reduced pressure in a vacuum oven at 110°C overnight prior to analysis.

### Data analysis

All data is reported as average ± standard deviation. All data points that are reported were calculated from *n* = 3, unless noted otherwise.

## Results

**FIGURE 4:**
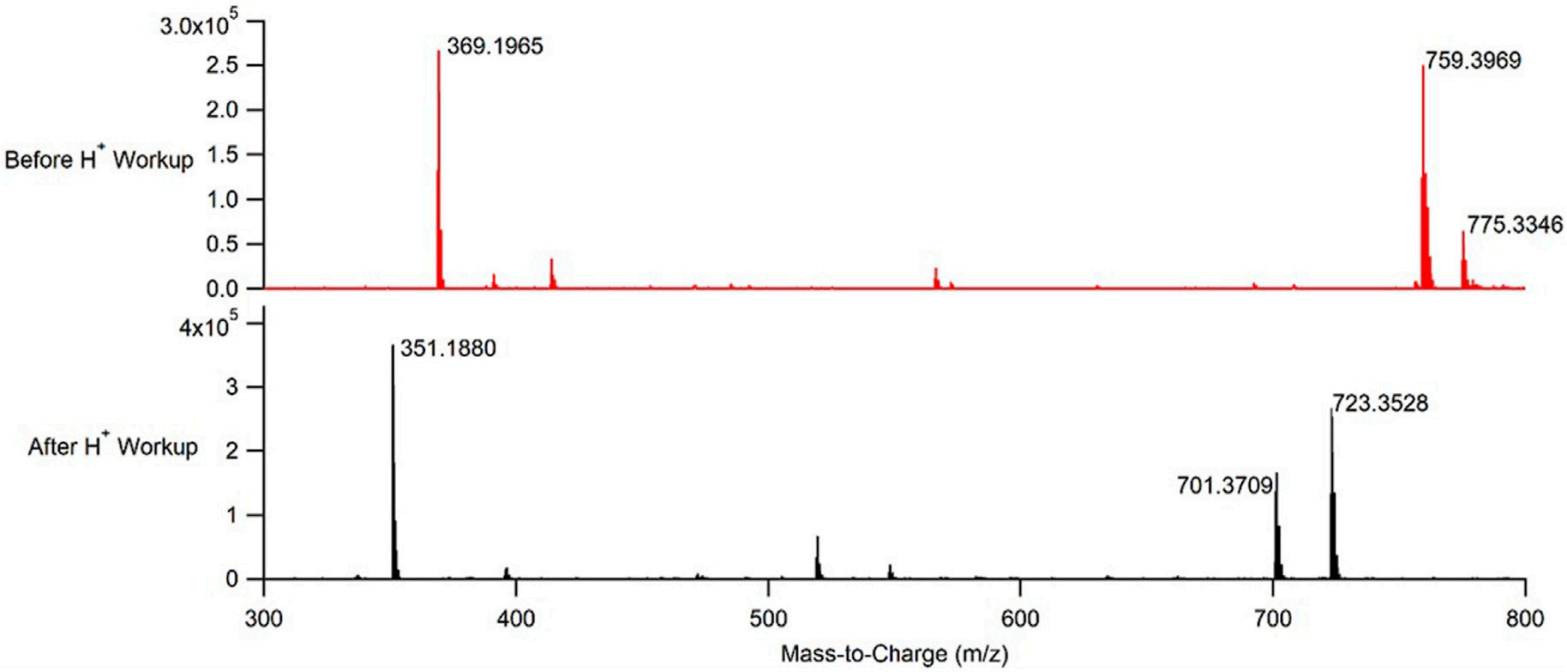
Representative mass spectra of the product obtained from performing CuBTC catalyzed condensations between benzaldehyde and dimedone under continuous flow conditions before an acid work-up (top, red), and after an acid work-up (bottom, black).

**FIGURE 5:**
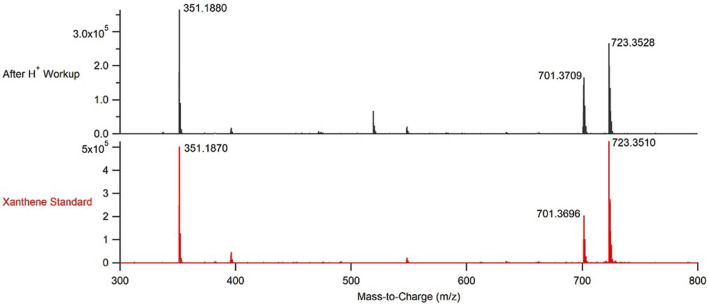
Representative mass spectrum of the product obtained from performing CuBTC catalyzed condensations between benzaldehyde and dimedone under continuous flow conditions after an acid work-up (top, black) and a mass spectrum of a purchased sample of the desired 3,3,6,6-tetramethyl-9-phenyl-3,4,5,6,7,9-hexahydro-1*H*-xanthene-1,8(2*H*)-dione (bottom, red).

The reaction to produce the 3,3,6,6-tetramethyl-9-phenyl-3,4,5,6,7,9-hexahydro-1*H*-xanthene-1,8 (2*H*)-dione xanthene derivative was performed under both batch and continuous flow reaction conditions. Notably, even though the CuBTC used to catalyze the reaction was directly packed into a stainless-steel column, there was no measured increase in pressure within the flow system at any point of running the reaction to produce **2**. The products from both reactions were subsequently analyzed using NMR, IR, and time-of-flight mass spectrometry (TOF-MS). Mass spectra of authentic samples of both the reactants and the product were also obtained (Supplementary Figures S42, S44, S45). Based on the TOF-MS data (Figures 4, 5; Supplementary Figures S3–S41), the continuous flow process used to synthesize **2** was successful in producing the open chain form of the desired xanthene, **1**. The successful synthesis of **1** was consistent regardless of whether the reaction was performed under batch or continuous flow conditions. Generation of **2** from **1** required an acid work-up step for both processes.

The expected product was confirmed using NMR and IR data. NMR provided evidence that the open chain form of the xanthene derivative was produced, having all of the expected peaks that would be expected from the open chain form of 3,3,6,6-tetramethyl-9-phenyl-3,4,5,6,7,9-hexahydro-1*H*-xanthene-1,8(2*H*)-dione product (^1^H NMR (CDCl_3_) δ 1.13 (s, 6H), 1.26 (s, 6H), 2.43 (m, 8H),5.57 (s, 1H), 7.18 (m, 2H), 7.29 (m, 3H)). The successful reaction to produce the open chain form of the product is further supported by the IR data where signals can be seen just under 3,000 cm^−1^, indicating the presence of sp^3^ C-H bonds and a peak around 1,650 cm^−1^ indicative of the presence of the C=O double bonds.

Based on the data obtained from SEM, pXRD, FT-IR, elemental analysis, and XPS evidence suggests that the CuBTC catalyst was not changing structurally under either batch or continuous flow conditions. After 10 separate 30 mL injections of the reaction flowed through the CuBTC column, the CuBTC particles remained intact based on the data below (*vide infra*). SEM images visually confirmed that the CuBTC particles remained intact. The pXRD data showed the retention of the crystal structure after the end of the reaction. The FT-IR spectroscopic data verified that there was no product adsorbed onto the MOF and that the ligand was still properly attached to the MOF.

**FIGURE 6:**
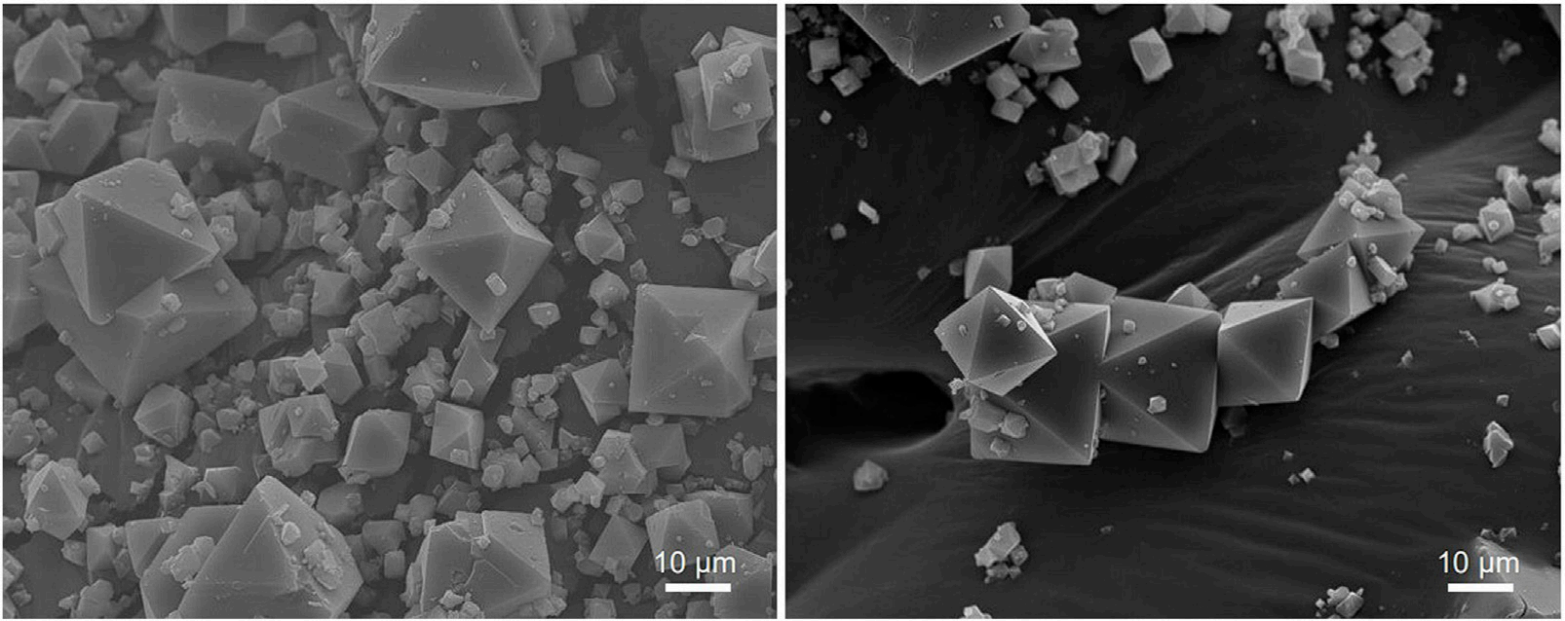
SEM images at ×1,000 magnification of CuBTC MOF before it has been used for catalysis (left) and after the CuBTC was used to catalyze condensations between benzaldehyde and dimedone under continuous flow conditions (right).

Furthermore, the SEM images of the CuBTC show well-defined octahedral particles mostly ranging from approximately 5–30 µm across the horizontal edge of the octahedra. Based on the SEM images obtained of the CuBTC before and after reaction (Figure 6), it can be seen that the MOF particles are visibly intact with no observed damage. The MOF particles also maintain their particle morphology and size, suggesting that CuBTC does not degrade under the reaction conditions.

**FIGURE 7:**
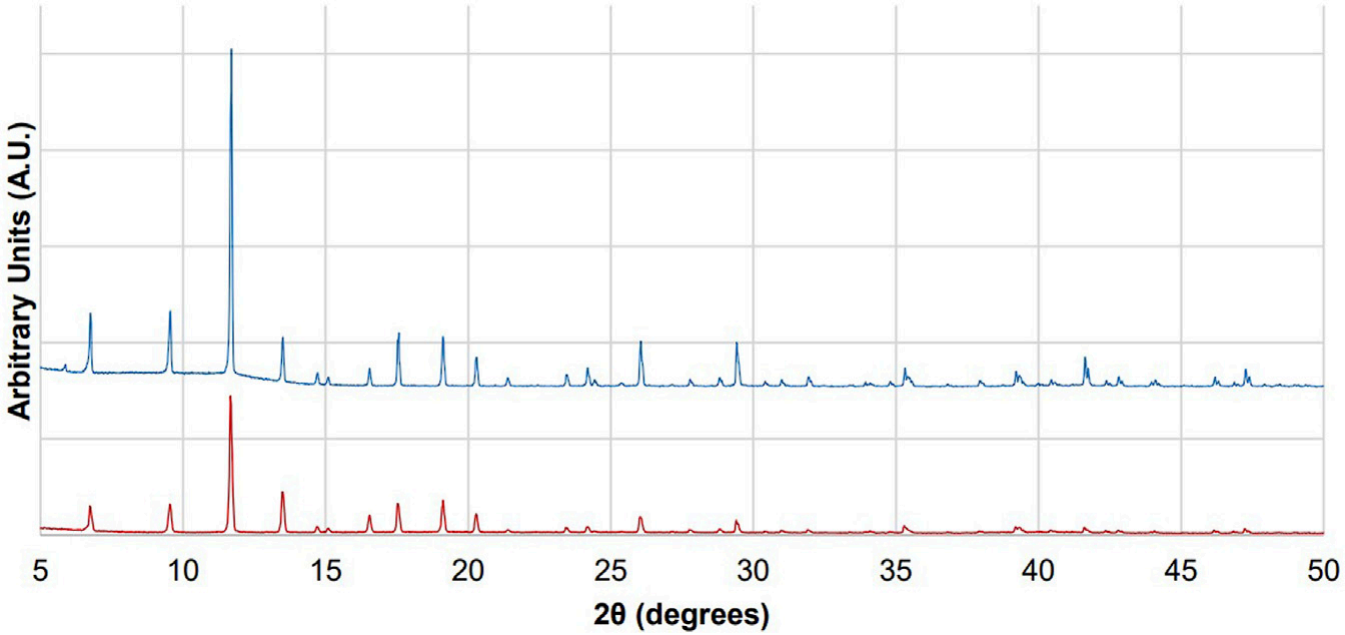
Diffraction patterns of CuBTC before it has been used for catalysis (top, blue) and after the CuBTC was used to catalyze condensations between benzaldehyde and dimedone (bottom, red).

The synthesized CuBTC yields a diffraction pattern (Figure 7) that matches the diffraction pattern as seen in the literature. (Neufeld et al., 2015; Schäfer et al., 2017; Peterson et al., 2019). The diffraction patterns obtained for CuBTC before and after continuous flow experiments (Figure 7) show that the diffraction peaks distinct and sharp after the reaction, consistent with a high degree of crystallinity in both samples. Furthermore, the position of the peaks does not change from the diffraction patterns taken before and after the CuBTC is used as a catalyst, supporting the hypothesis that the bulk crystal structure of CuBTC does not change over the course of the reaction.

**FIGURE 8:**
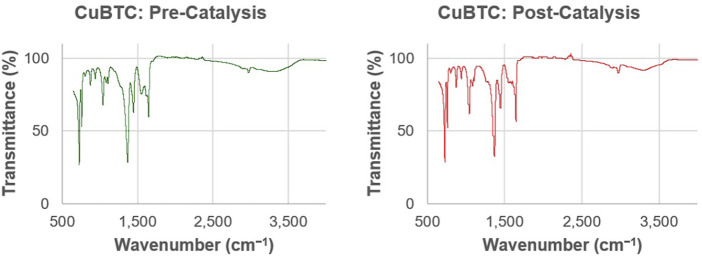
Representative FT-IR spectra of CuBTC before (left, green) and CuBTC after (right, red) it was used to catalyze the condensation reactions under continuous flow to produce a xanthene derivative.

The FT-IR spectra of CuBTC (Figure 8) matches spectra as reported in previous literature. (Cai et al., 2020; Nivetha et al., 2020). Hydroxyl groups are indicated by the presence of stretching vibrations in the 3,000–3,500 cm^−1^ range. The peaks at 1,108, 1,370, and 1,650 cm^−1^ are indicative of asymmetric and symmetric carboxylate groups from the H_3_BTC ligand. Lastly, the peaks at 759 and 728 cm^−1^ are indicative of Cu-O linkages. The confirmed presence of the Cu-O bonds suggest that the copper centers are still attached to the H_3_BTC ligands. The peaks that appear in the IR spectra obtained before and after the CuBTC was used for catalysis are identical, indicating that there were no observed changes in the ligand-Cu bonds of the MOF as a result of being used to catalyze the reaction.

Based on the yield data, the same amount of product can be generated in less time under continuous flow conditions using a direct-packed CuBTC column. Yields were comparative between batch conditions vs. continuous flow conditions (33% ± 0.12%, *n* = 6 vs. 33% ± 14%, *n* = 27), but the time needed to perform the reaction and isolate the product is greatly decreased for continuous flow. Performing the reaction under batch conditions requires ∼15 min of reflux and catalyst recovery can require up to 2 h. However, under continuous flow conditions, the reaction flows through the system in 6 min and cleaning the column only requires 25 min in between injections. Based on these time points, performing the reaction under the continuous flow conditions outlined herein requires only about 23% of the time it would take to perform the reaction under batch conditions (i.e., it is approximately four times faster to use the continuous flow conditions than batch conditions). Additionally, in a true continuous flow system, the repeated 25-min wash in between each injection would not be necessary. With the need for repeated washing eliminated, the continuous flow would only take approximately 4% of the time necessary to produce the same amount of product as under batch conditions at this scale. Hence, the proposed upper limit for the continuous flow system is 22.5x faster than batch conditions.

To directly confirm the identity of the desired product and demonstrate that the MOF remained intact, several key characterization techniques were employed. First, elemental analysis was performed via ICP-HRMS (Supplementary Table S2). Starting materials (*N,N*-dimethylformamide and dimedone) and reaction solvent (ethanol) were used as controls. These controls, and the collected eluent were analyzed to determine the amount of free copper present in the samples. This analysis revealed that the product contained 3 ± 1 μg Cu per 1 g of sample. This is well below the approved 340 μg per day parenteral exposure limit advised by FDA Guidance Q3D (R2). (Nguyen et al., 2012). Based on previous literature, the numbers obtained from elemental analysis are likely indicative of the presence of residual extraframework monomeric Cu (II) ions and are not indicative of the CuBTC MOF degrading (Pöppl et al., 2008). As a final confirmation of the MOF structural stability, the MOF was analyzed via XPS to identify the oxidation states of the copper sites in the CuBTC MOF before and after the reaction. Comparisons of the XPS scans show that the oxidation numbers remain consistent before and after the reaction (Supplementary Figures S46–S51).

Additionally, TOF-MS and NMR were able to indirectly provide further evidence that the CuBTC used to catalyze the reaction remained intact under the reaction conditions. The natural isotopes of Cu, 62.9 amu and 64.9 amu, occur at abundances of 69% and 31% respectively. In mass spectrometry, if a compound or solution contains Cu, one would expect to see a peak at [M + 2] with a relative abundance of approximately 45% the height of the molecular ion [M] (Tsednee et al., 2016). 36 This peak was not observed in our mass spectra, supporting our claim that our synthesized products do not contain copper. Cu^2+^ is also paramagnetic, which affects both the chemical shift and relaxation time of protons when studying via NMR. The paramagnetic effect of Cu^2+^ causes line broadening of the observed spectra (Nguyen et al., 2012). No such broadening was observed, which also supports our claim that the synthesized products do not contain copper. With the data from the TOF-MS in combination with the SEM, pXRD, FT-IR, elemental analysis, and XPS data, it can be concluded that CuBTC is catalytic and does not change structurally with continuous use; thus strongly suggesting that the CuBTC in the column has the potential for infinite use.

## Conclusion

In this work, CuBTC was successfully used to catalyze the reaction between dimedone and benzaldehyde to produce a 1,8-dioxo-octa-hydro-xanthene derivative under continuous flow conditions. Using continuous flow to catalyze the production of a xanthene derivative was shown to be more efficient than published batch conditions, producing a xanthene derivative 22.5x faster at comparable yields. This increased efficiency is due to the lower reaction time required (6 min of flow under continuous flow compared with 15 min of reflux under batch conditions) and because operating under continuous flow allows one to forgo catalyst isolation and recovery steps. The continuous flow system generated the desired product while maintaining the mild reaction conditions, use of non-toxic solvents, and producing comparable yields to batch. Based on SEM, pXRD, IR, and elemental analysis data collected, the CuBTC was demonstrated to be stable under the reaction conditions with no observable changes or damages to the MOF particles, crystallinity of the MOF, or functional groups. Moving forward, scaling up the continuous flow system by using larger catalytic columns and higher flow rates is desirable to further optimize reaction time and performance. Furthermore, a scope study to investigate the potential of the continuous flow system to produce a larger variety of xanthene derivatives would be of high importance. Identifying how the presence of electron withdrawing and electron donating groups have on the efficiency of the continuous flow system would be crucial in improving this continuous flow system. Additionally, the required acid work-up could potentially be built into the continuous flow system itself, allowing for the entire synthetic process to be automated.

## Data Availability

The original contributions presented in the study are included in the article/Supplementary Material, further inquiries can be directed to the corresponding author.
