# Supplementary material for: Continuous flow catalysis with CuBTC improves reaction time for synthesis of xanthene derivatives
Source: Front Chem. 2023 Oct 16;11:1259835. doi: 10.3389/fchem.2023.1259835 (PMC10613637; doi:10.3389/fchem.2023.1259835)
Supplement: Supplementary file 1 [file DataSheet1.PDF]

Supporting Information

Continuous Flow Catalysis with CuBTC Improves Reaction Time for Synthesis of Xanthene Derivatives

Jonathan E. Thai [a], *Madeline C. Roach* [a], *Melissa M. Reynolds* [*a,b,c]

[a] Department of Chemistry, Colorado State University, 1801 Campus Delivery, Fort Collins, Colorado 80523, United States

[b] School of Biomedical Engineering, Colorado State University, 1376 Campus Delivery, Fort Collins, Colorado 80523, United States

[c] Department of Chemical and Biological Engineering, Colorado State University, 1370 Campus Delivery, Fort Collins, Colorado 80523, United States

Corresponding Author:

* M. Reynolds. Email: Melissa.Reynolds@colostate.edu

Table of Contents

Page Number

Representative NMR of product S3

Representative IR of product S4

Synthetic Conditions Used for Batch Conditions S5

Comparison Between Batch Conditions and Continuous Flow System S6

Mass Spectra of Products from Batch Conditions S7

Mass Spectra of Products from Continuous Flow System S14

Mass Spectrum of Purchased Standard Product S46

Mass Spectrum of Product Synthesized Using Continuous Flow System After H^+ Workup S47

Mass Spectra of Starting Materials S48

Elemental Analysis Data Summary S50

XPS Data S51

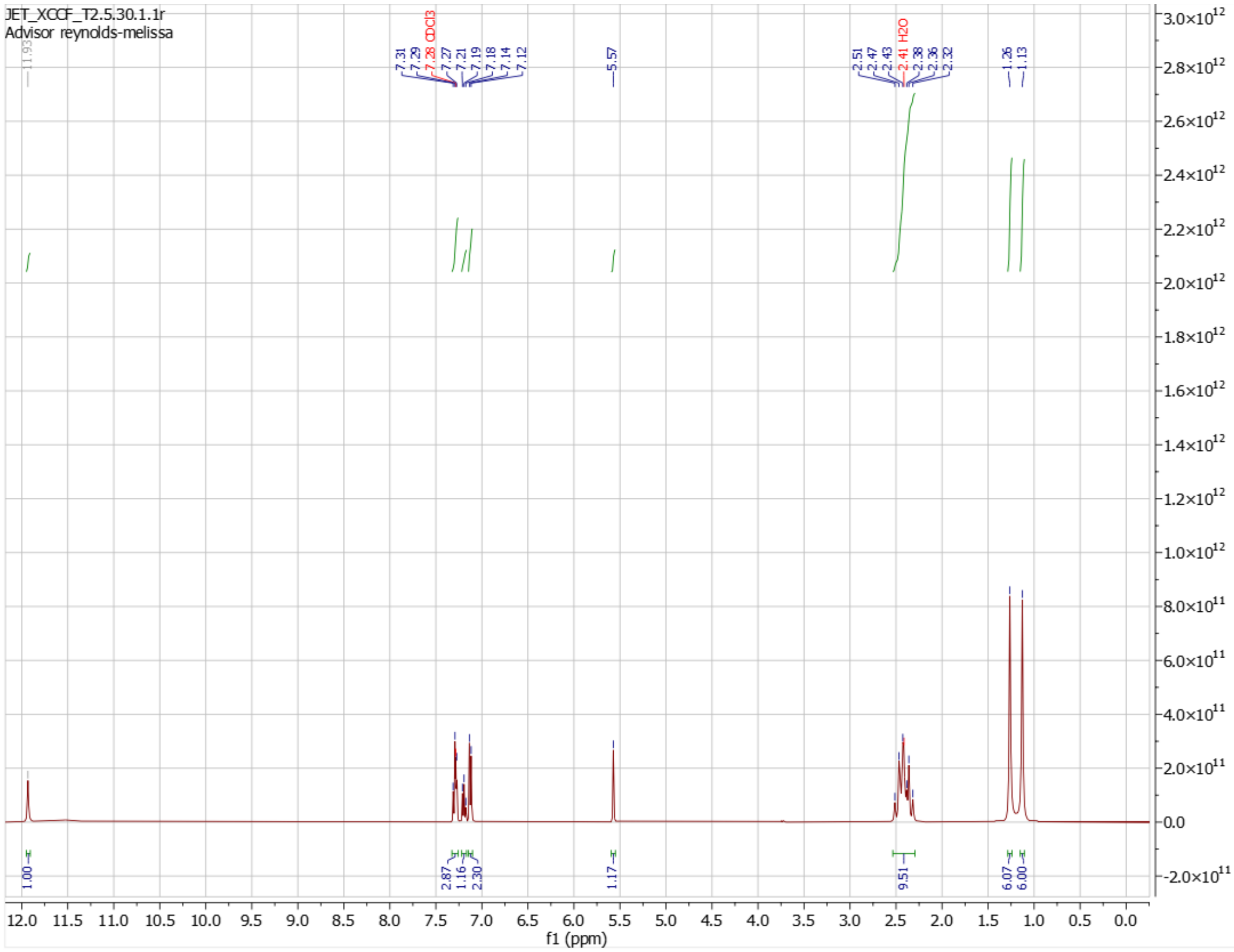

Figure S1. Representative NMR of open chain form of 3,3,6,6-tetramethyl-9-phenyl-3,4,5,6,7,9-hexahydro-1*H*-xanthene-1,8(2*H*)-dione in $CDCl_3$.

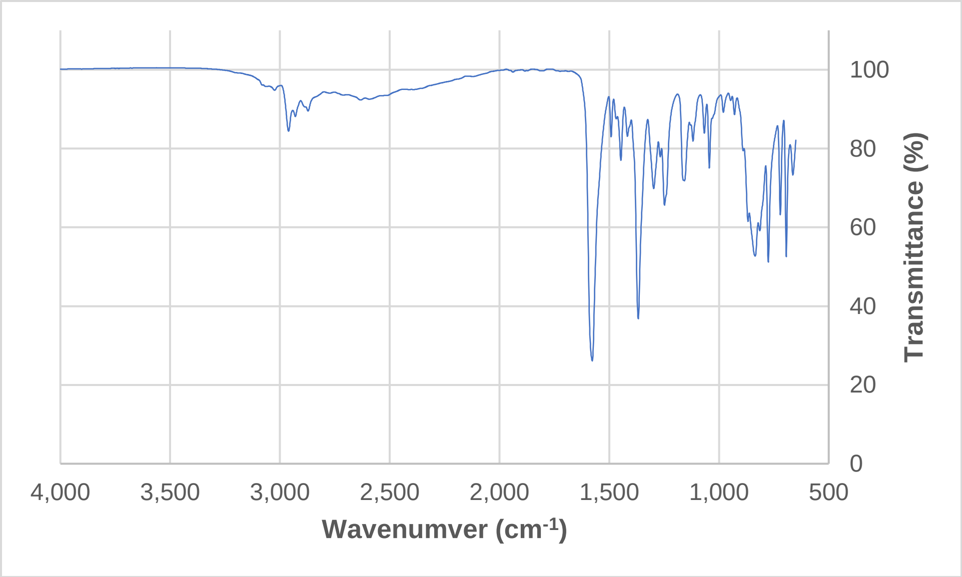

Figure S2. Representative IR of the open chain form of 3,3,6,6-tetramethyl-9-phenyl-3,4,5,6,7,9-hexahydro-1*H*-xanthene-1,8(2*H*)-dione.

Using CuBTC to Catalyze the Condensation Between Dimedone and Benzaldehyde Under Batch Conditions:

The reaction conditions used were adapted from the literature.[14] In short, CuBTC (0.05 g, 0.083 mmol) was weighed out into a 50 mL round bottomed flask. The CuBTC was then activated in a vacuum oven at 150°C under reduced pressure overnight. The oven was then turned off and the CuBTC was then allowed to cool naturally to room temperature before it was taken out. Dimedone (2.0 mmol, 2 equiv.) and benzaldehyde (1.0 mmol, 1 equiv.) were measured out and added to the flask containing the activated CuBTC. Ethanol (3 mL) was then measured out and added to the RBF. The reaction was then stirred and heated to 80°C for 20 minutes in a reflux apparatus. Once completed, the reaction mixture was centrifuged to separate the CuBTC from the crude product. Solvent was removed from the crude product under reduced pressure, producing a white powder. The solids were then recrystallized to produce white/clear rectangular crystals. The resulting products were then analyzed using TOF-MS.

To determine the increase in efficiency, approximated average times were used for each of the steps that were improved upon (Table S1) and the equations used are shown below. These calculations are also assuming one is not washing the column after every 3 mL of reactant mixture as it would not be necessary in a true continuous flow system where the reaction mixture is constantly flowing through the system. Based on these calculations, the continuous flow setup used could produce the desired xanthene derivative at 22.5x the rate it could be produced under batch conditions when comparing reaction mixture volumes of 3 mL and using a 0.5 mL/minute flow rate on the continuous flow system.

Table S1.

	**Reaction Time**	**Catalyst Recovery**	**Total Time**
**Batch Conditions**	15 minutes	120 minutes	135 minutes
**Continuous Flow**	6 minutes	0 minutes	6 minutes

Equation S1.

$$\frac{135\ minutes}{6\ minutes} = 22.5$$

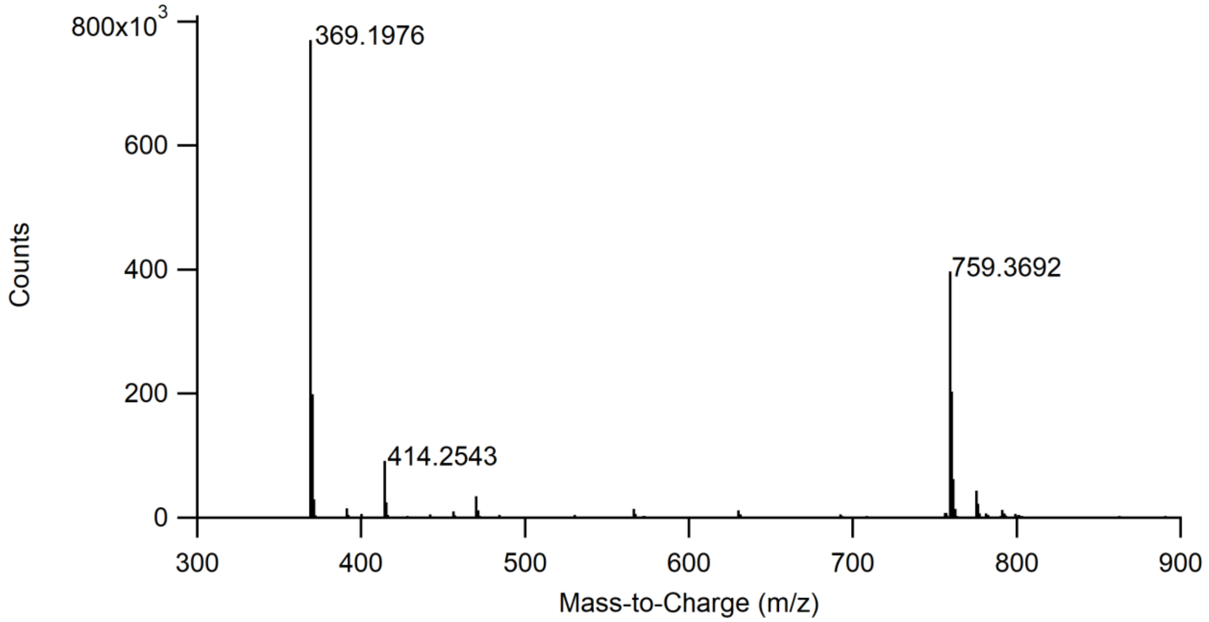

Figure S3. Mass spectrum of the open-chain form of 3,3,6,6-tetramethyl-9-phenyl-3,4,5,6,7,9-hexahydro-1*H*-xanthene-1,8(2*H*)-dione synthesized using batch conditions using the method as reported by Ghafuri et al. at 75°C.

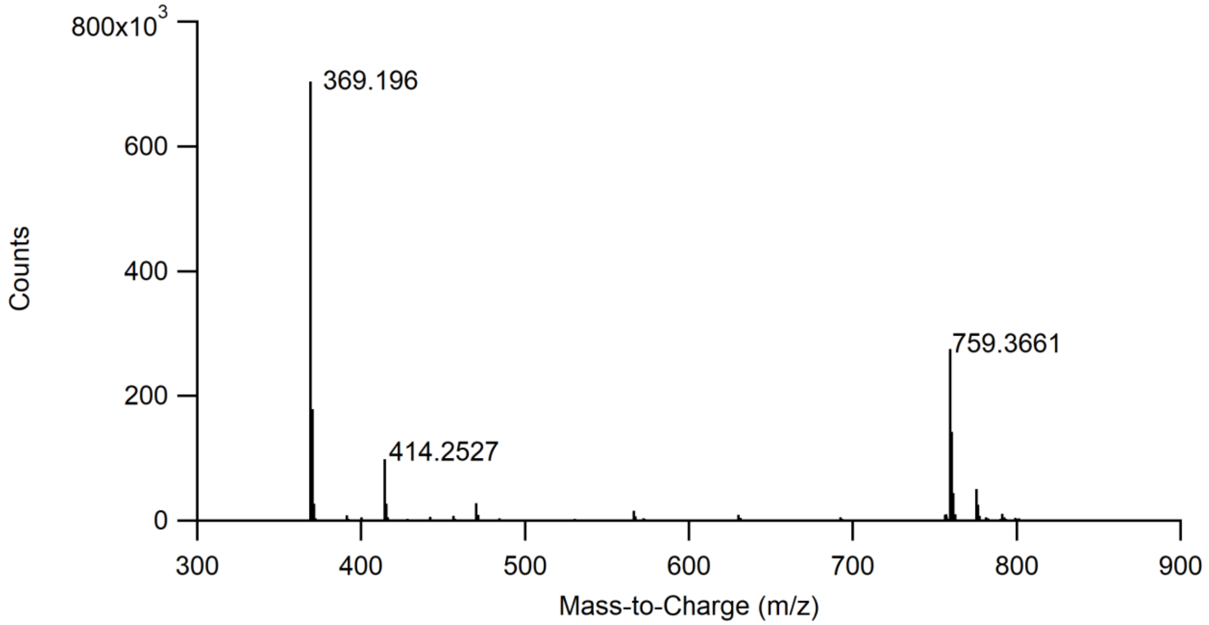

Figure S4. Mass spectrum of the open-chain form of 3,3,6,6-tetramethyl-9-phenyl-3,4,5,6,7,9-hexahydro-1*H*-xanthene-1,8(2*H*)-dione synthesized using batch conditions using the method as reported by Ghafuri et al. at 80°C.

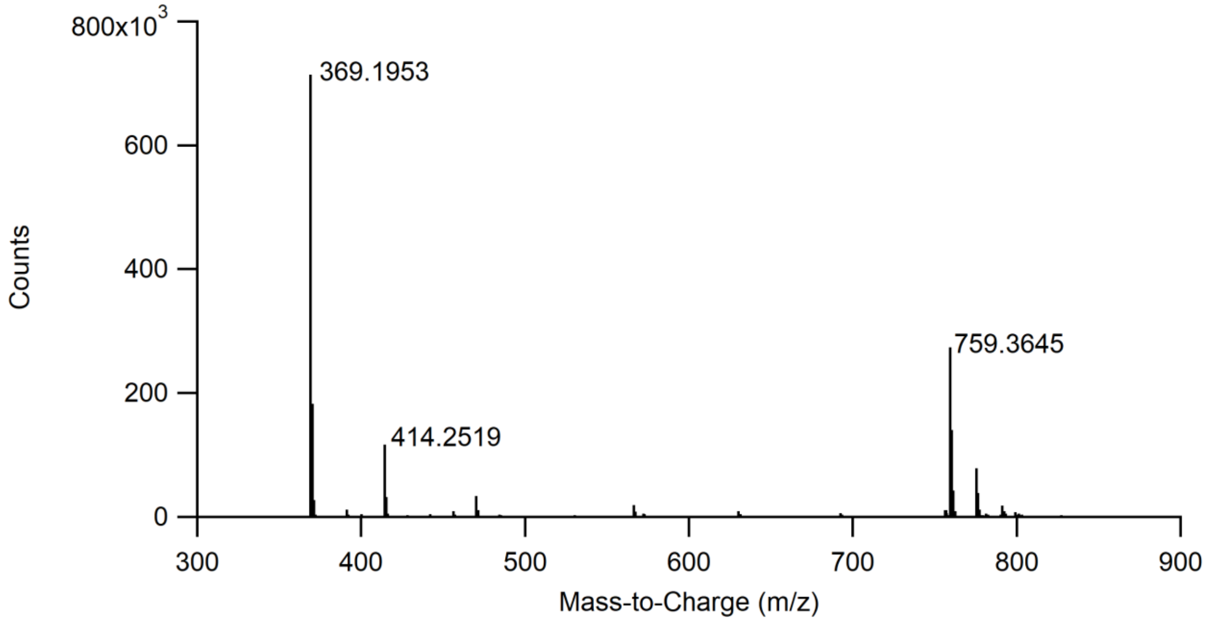

Figure S5. Mass spectrum of the open-chain form of 3,3,6,6-tetramethyl-9-phenyl-3,4,5,6,7,9-hexahydro-1*H*-xanthene-1,8(2*H*)-dione synthesized using batch conditions using the method as reported by Ghafuri et al. at 85°C.

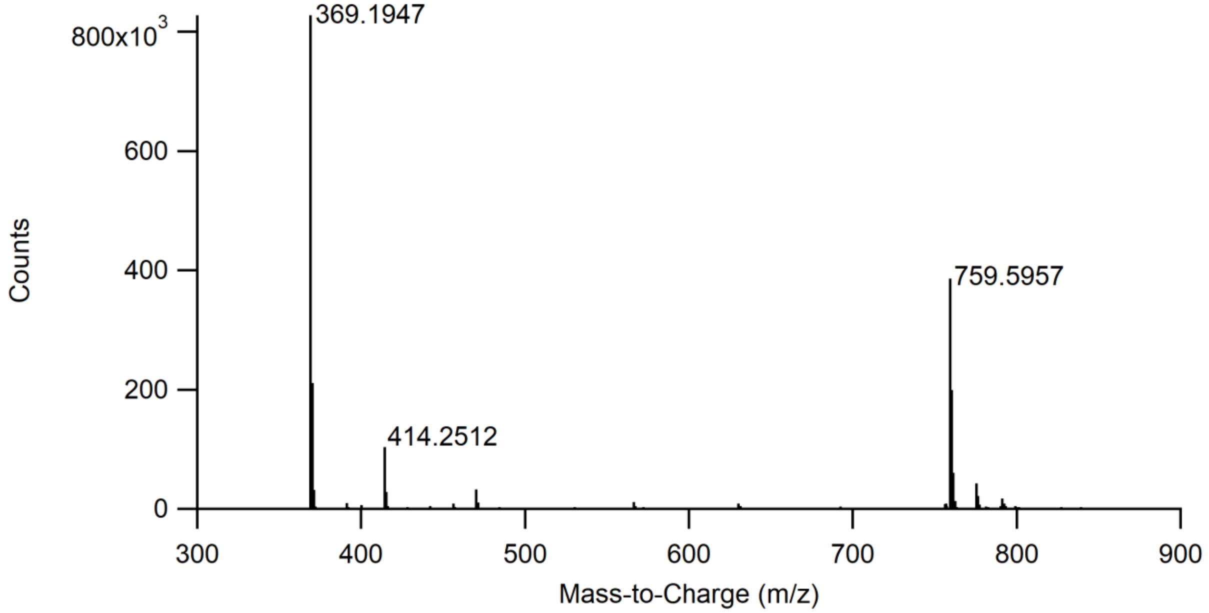

Figure S6. Mass spectrum of the open-chain form of 3,3,6,6-tetramethyl-9-phenyl-3,4,5,6,7,9-hexahydro-1*H*-xanthene-1,8(2*H*)-dione synthesized using batch conditions using the method as reported by Ghafuri et al. at 90°C.

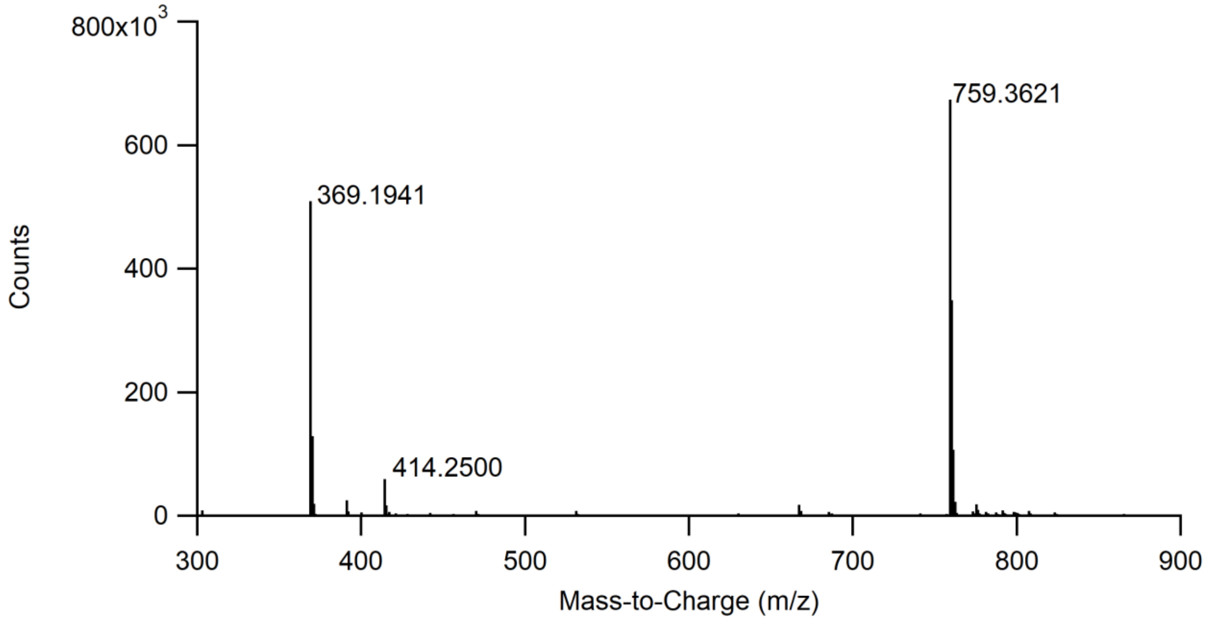

Figure S7. Mass spectrum of the open-chain form of 3,3,6,6-tetramethyl-9-phenyl-3,4,5,6,7,9-hexahydro-1*H*-xanthene-1,8(2*H*)-dione synthesized using batch conditions using the method as reported by Ghafuri et al. at 80°C (as a second trial).

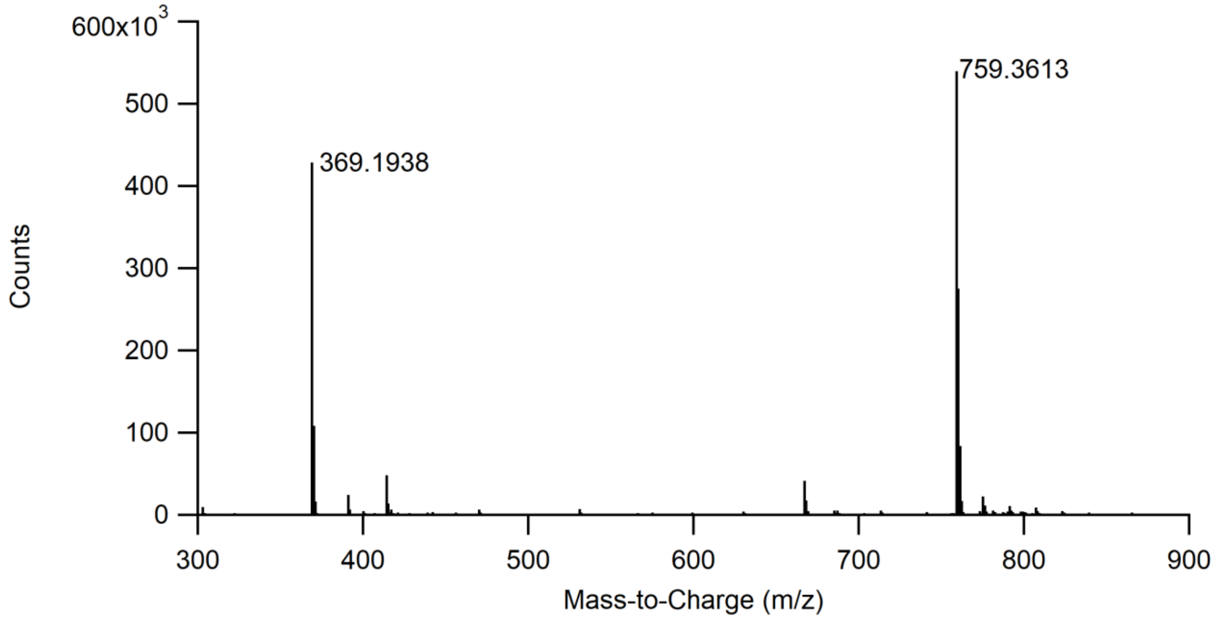

Figure S8. Mass spectrum of the open-chain form of 3,3,6,6-tetramethyl-9-phenyl-3,4,5,6,7,9-hexahydro-1*H*-xanthene-1,8(2*H*)-dione synthesized using batch conditions using the method as reported by Ghafuri et al. at 80°C (as a third trial to obtain data for batch conditions at 80°C with n = 3).

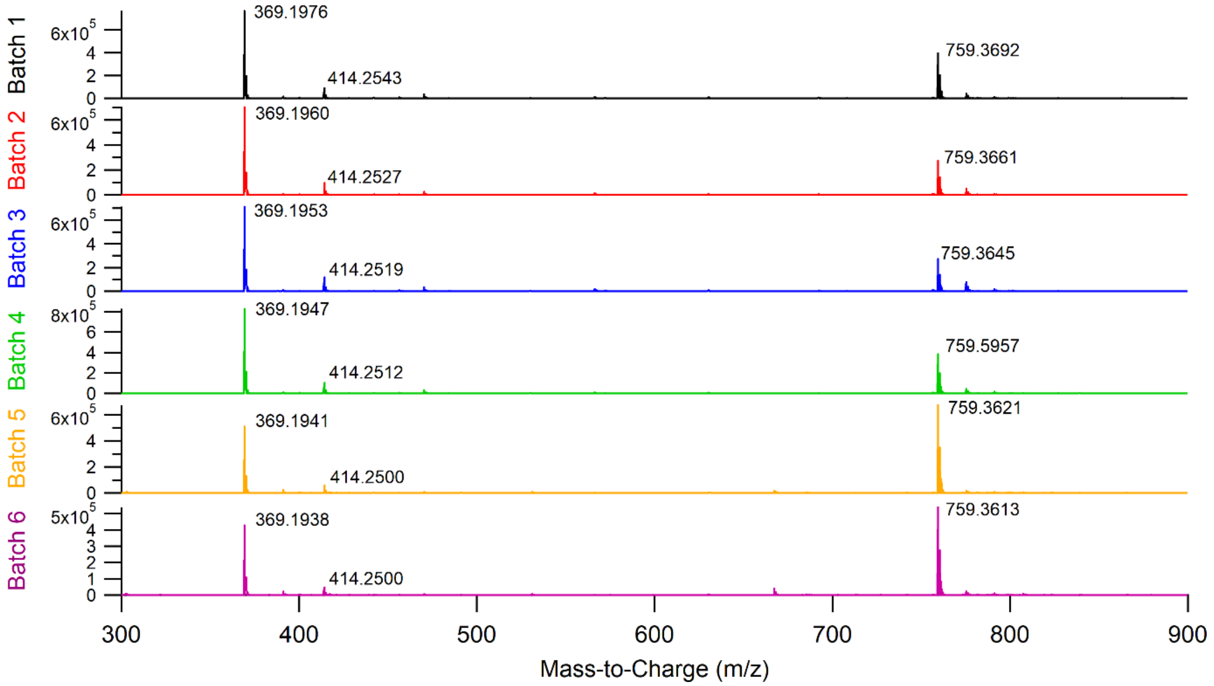

Figure S9. All of the mass spectra obtained of the open-chain form of 3,3,6,6-tetramethyl-9-phenyl-3,4,5,6,7,9-hexahydro-1*H*-xanthene-1,8(2*H*)-dione synthesized using batch conditions using the method as reported by Ghafuri et al. stacked. For reference, Batch 1 = Figure S3, Batch 2 = Figure S4, Batch 3 = Figure S5, Batch 4 = Figure S6, Batch 5 = Figure S7, and Batch 6 = Figure S8.

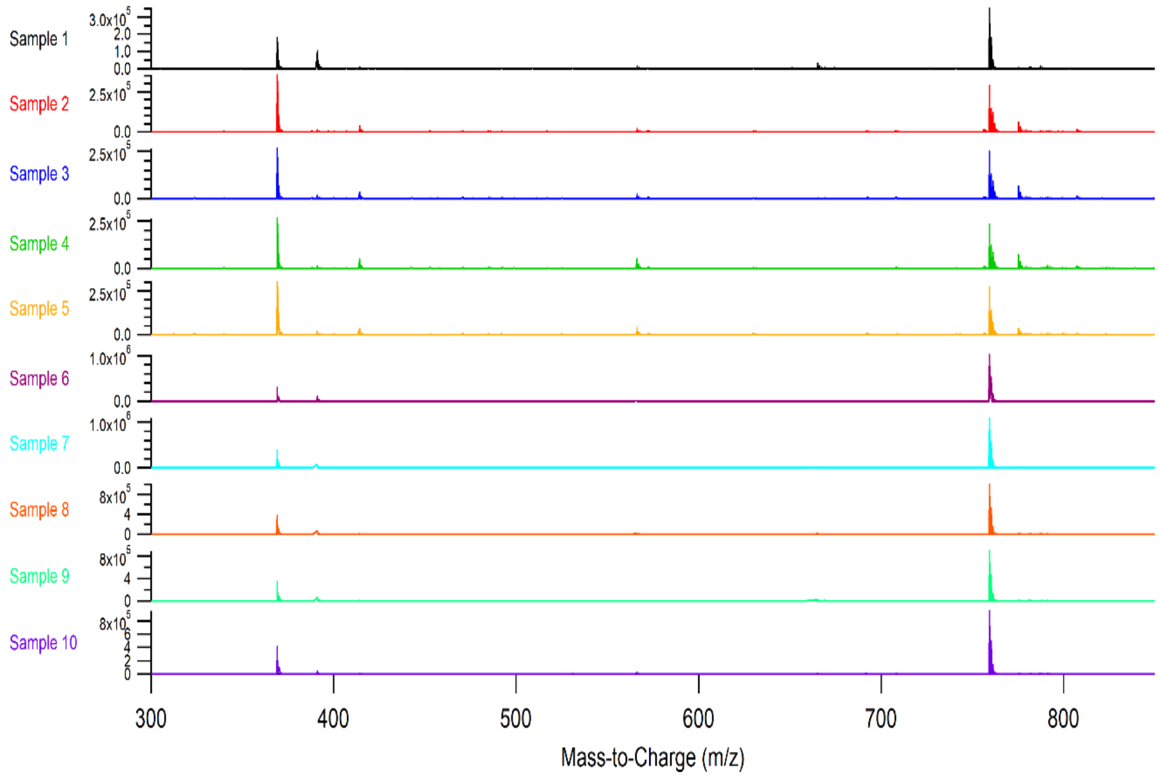

Figure S10. All of the mass spectra obtained of the open-chain form of 3,3,6,6-tetramethyl-9-phenyl-3,4,5,6,7,9-hexahydro-1*H*-xanthene-1,8(2*H*)-dione synthesized using the continuous flow reactor using the same column for all samples. Between each 3 mL injection of reaction mixture, 25 mL of ethanol was used to rinse out the tubing and column on the reactor. Each "sample" is representative of the product collected from each injection after it had excess solvent removed and recrystallized. This includes data from Figures S11 – S20.

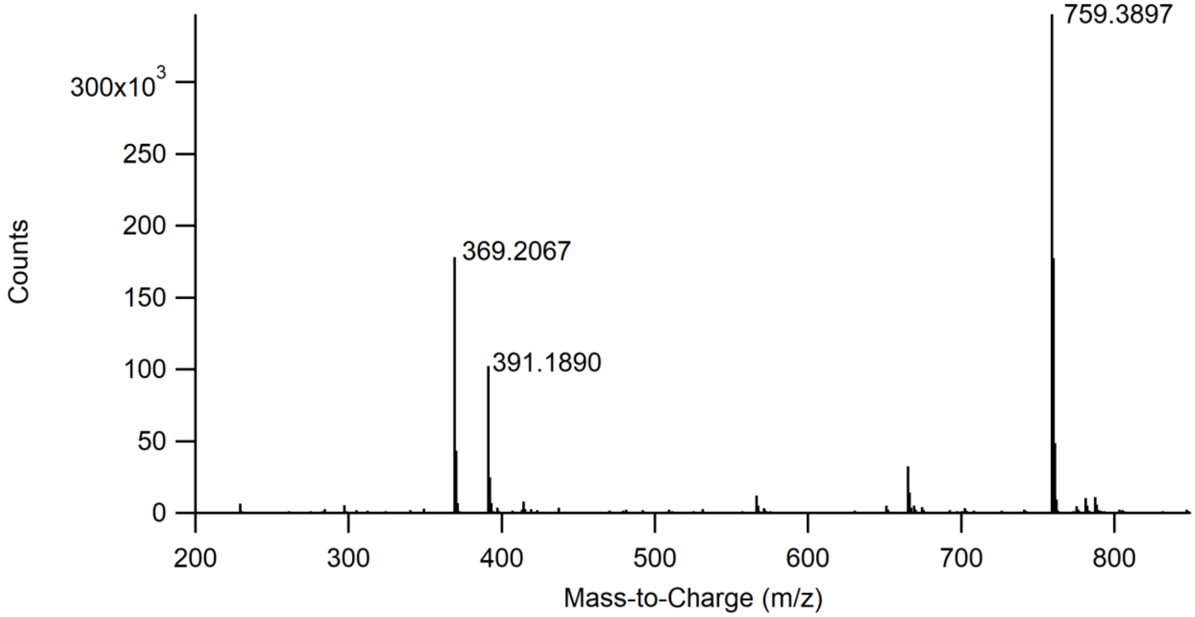

Figure S11. Mass spectrum obtained of the open-chain form of 3,3,6,6-tetramethyl-9-phenyl-3,4,5,6,7,9-hexahydro-1*H*-xanthene-1,8(2*H*)-dione synthesized using the continuous flow reactor. This was sampled from the first 3 mL of reaction mixture to flow through this column.

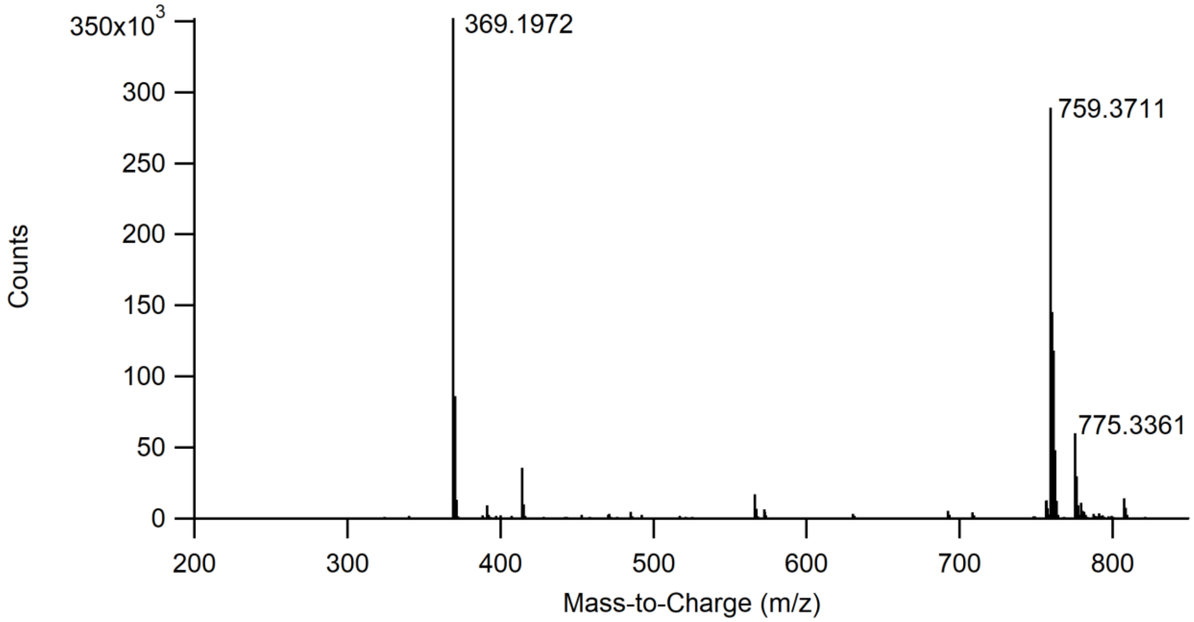

Figure S12. Mass spectrum obtained of the open-chain form of 3,3,6,6-tetramethyl-9-phenyl-3,4,5,6,7,9-hexahydro-1*H*-xanthene-1,8(2*H*)-dione synthesized using the continuous flow reactor. This was sampled from the second 3 mL of reaction mixture to flow through this column.

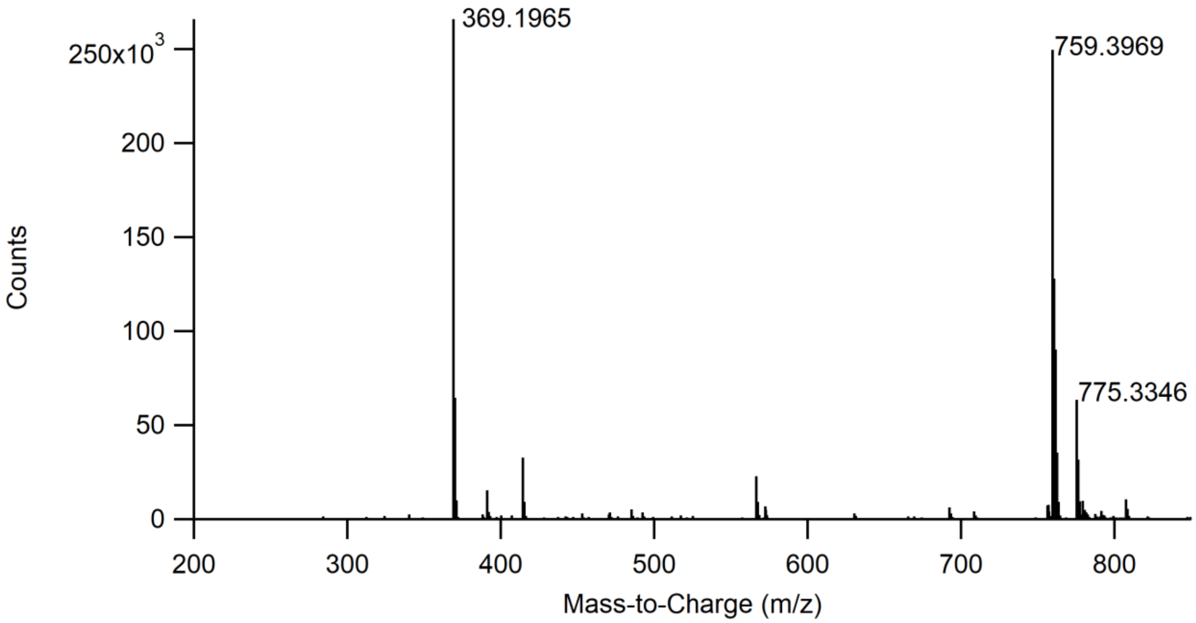

Figure S13. Mass spectrum obtained of the open-chain form of 3,3,6,6-tetramethyl-9-phenyl-3,4,5,6,7,9-hexahydro-1*H*-xanthene-1,8(2*H*)-dione synthesized using the continuous flow reactor. This was sampled from the third 3 mL of reaction mixture to flow through this column.

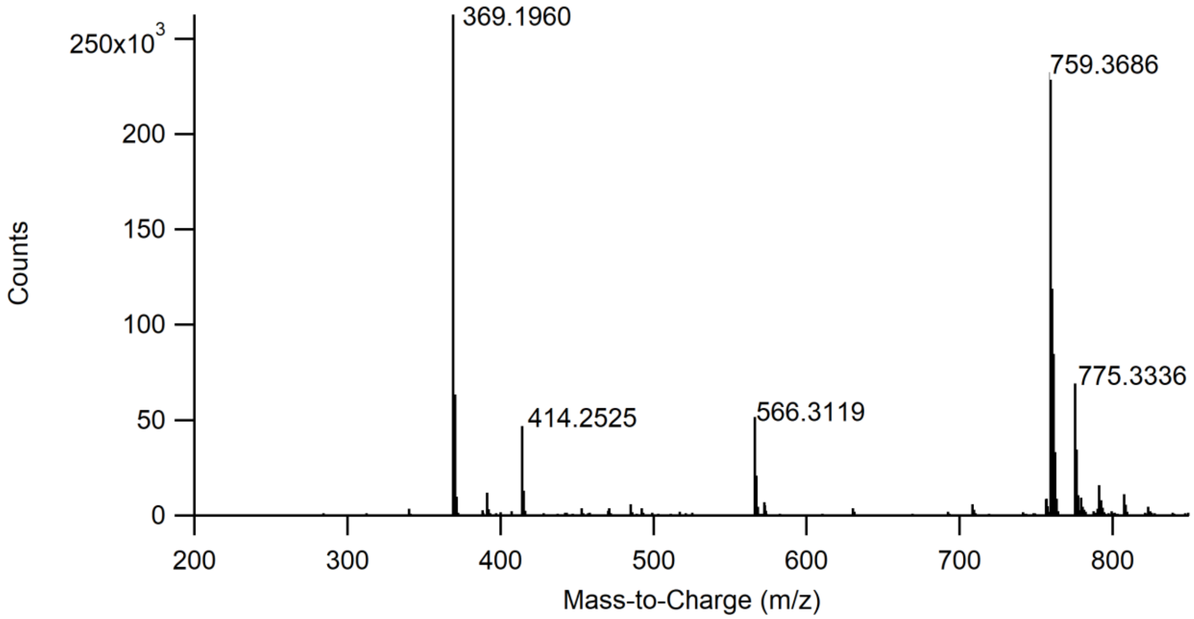

Figure S14. Mass spectrum obtained of the open-chain form of 3,3,6,6-tetramethyl-9-phenyl-3,4,5,6,7,9-hexahydro-1*H*-xanthene-1,8(2*H*)-dione synthesized using the continuous flow reactor. This was sampled from the fourth 3 mL of reaction mixture to flow through this column.

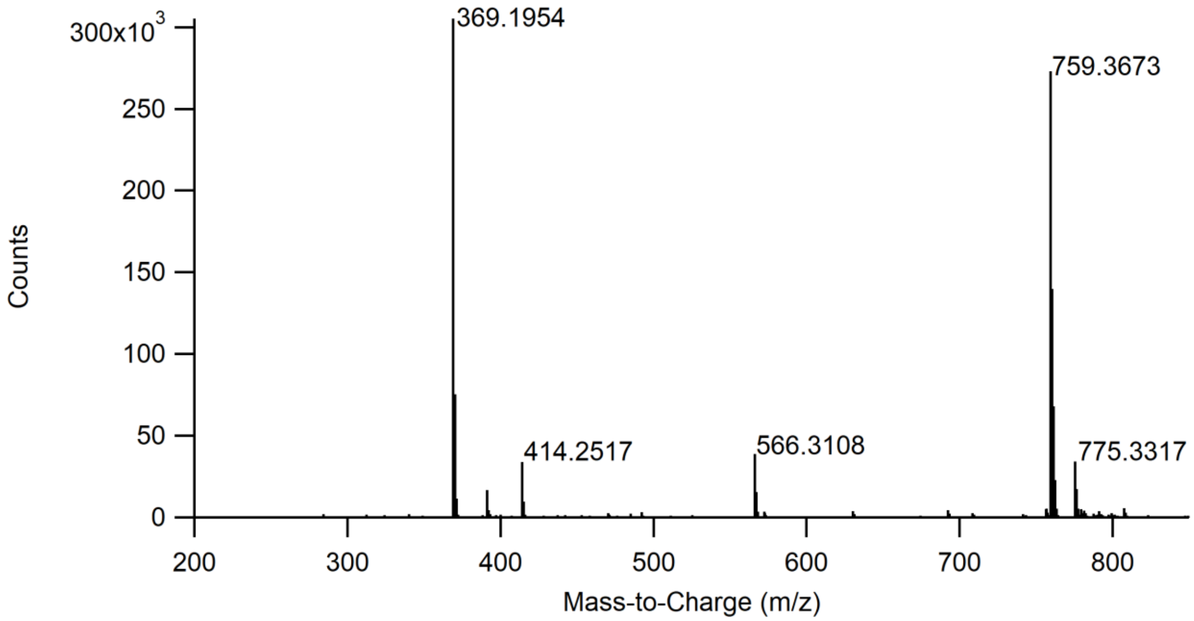

Figure S15. Mass spectrum obtained of the open-chain form of 3,3,6,6-tetramethyl-9-phenyl-3,4,5,6,7,9-hexahydro-1*H*-xanthene-1,8(2*H*)-dione synthesized using the continuous flow reactor. This was sampled from the fifth 3 mL of reaction mixture to flow through this column.

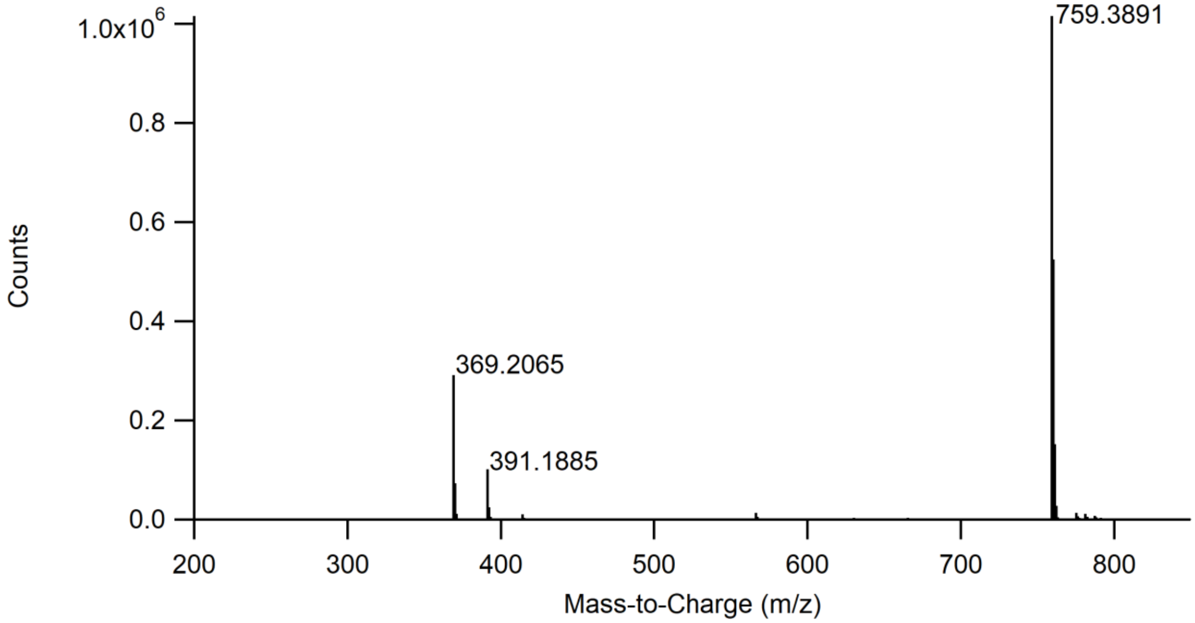

Figure S16. Mass spectrum obtained of the open-chain form of 3,3,6,6-tetramethyl-9-phenyl-3,4,5,6,7,9-hexahydro-1*H*-xanthene-1,8(2*H*)-dione synthesized using the continuous flow reactor. This was sampled from the sixth 3 mL of reaction mixture to flow through this column.

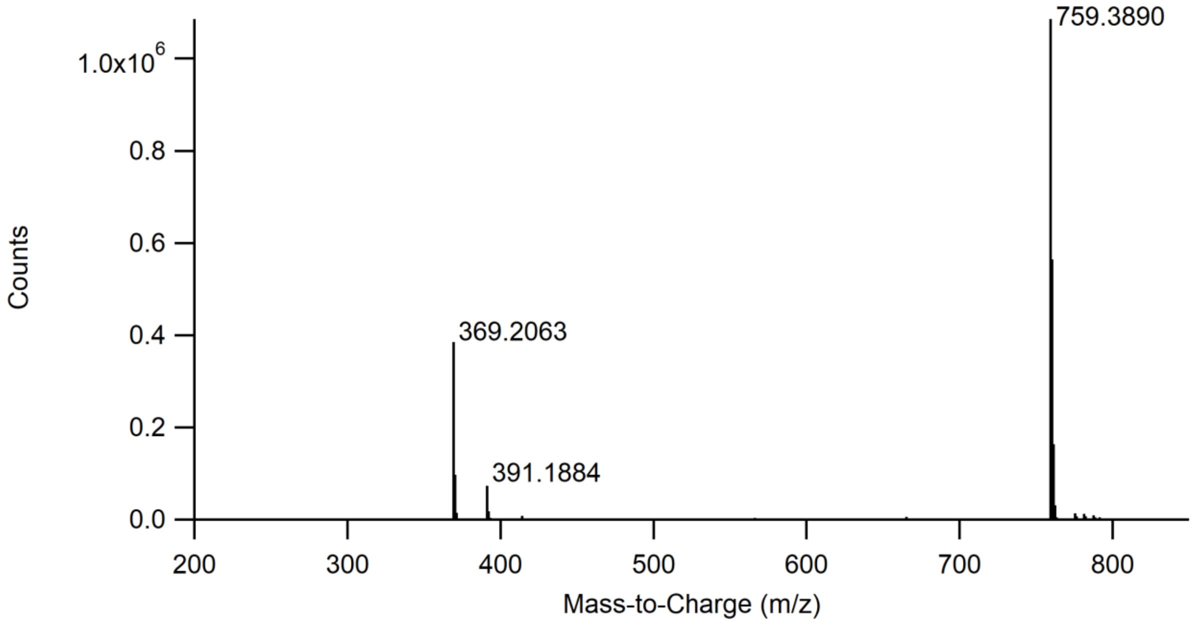

Figure S17. Mass spectrum obtained of the open-chain form of 3,3,6,6-tetramethyl-9-phenyl-3,4,5,6,7,9-hexahydro-1*H*-xanthene-1,8(2*H*)-dione synthesized using the continuous flow reactor. This was sampled from the seventh 3 mL of reaction mixture to flow through this column.

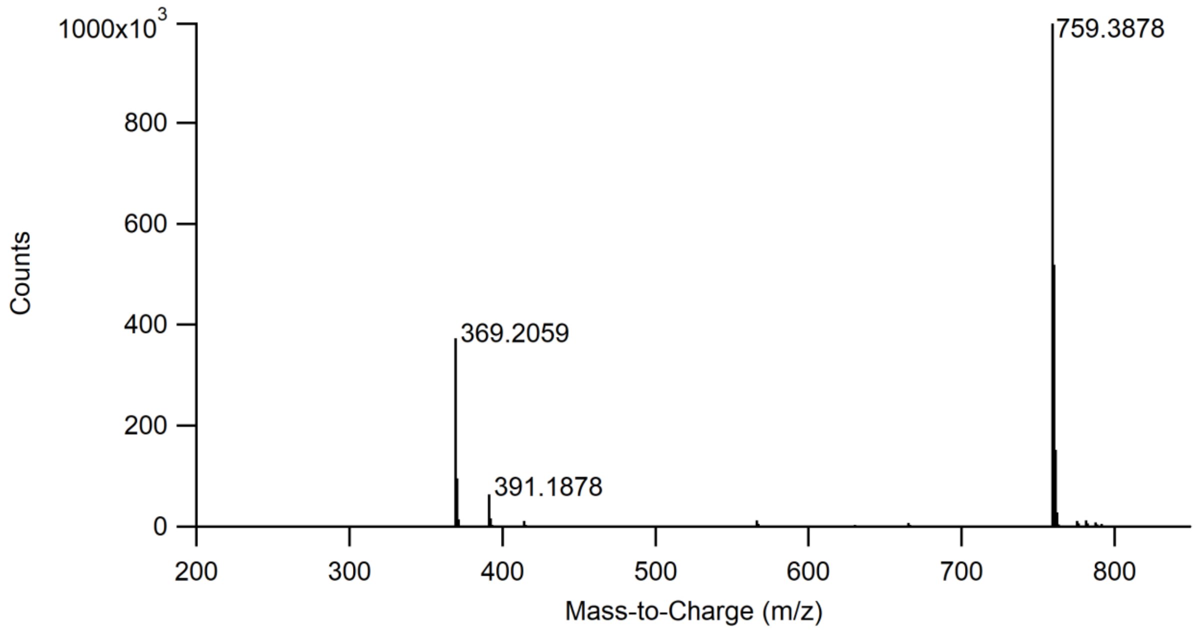

Figure S18. Mass spectrum obtained of the open-chain form of 3,3,6,6-tetramethyl-9-phenyl-3,4,5,6,7,9-hexahydro-1*H*-xanthene-1,8(2*H*)-dione synthesized using the continuous flow reactor. This was sampled from the eighth 3 mL of reaction mixture to flow through this column.

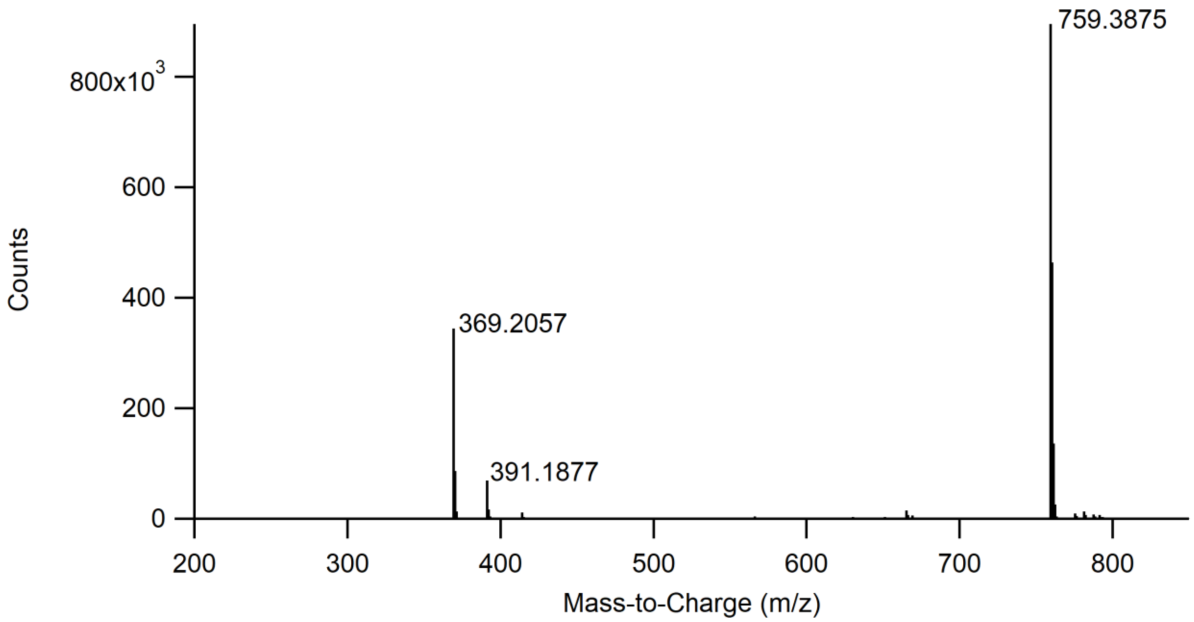

Figure S19. Mass spectrum obtained of the open-chain form of 3,3,6,6-tetramethyl-9-phenyl-3,4,5,6,7,9-hexahydro-1*H*-xanthene-1,8(2*H*)-dione synthesized using the continuous flow reactor. This was sampled from the ninth 3 mL of reaction mixture to flow through this column.

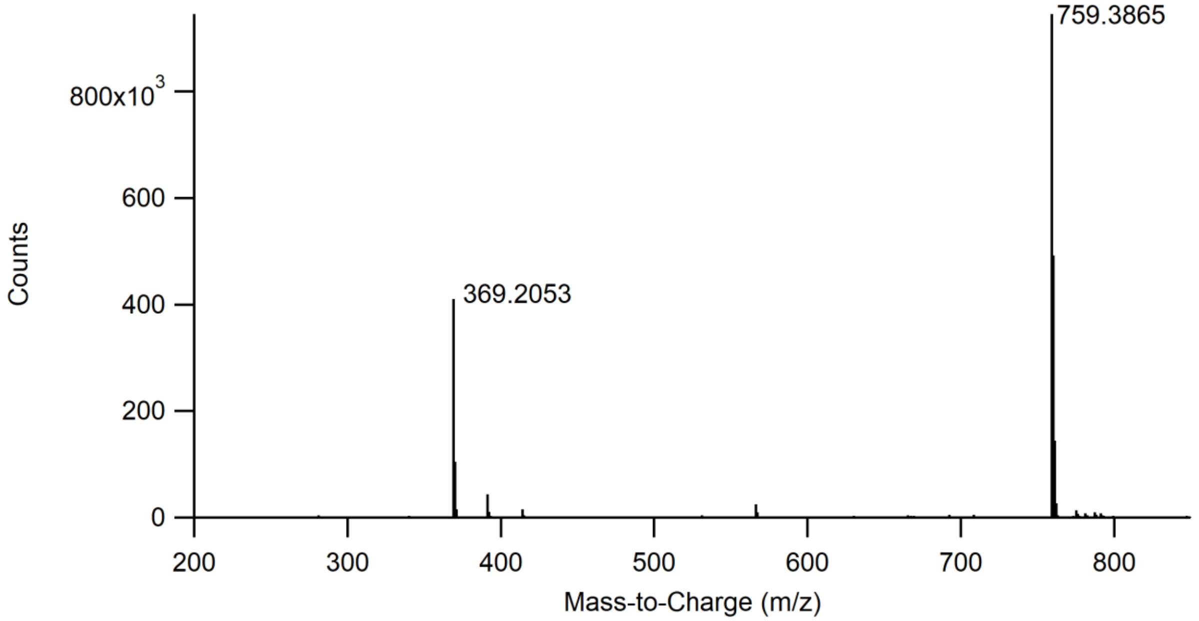

Figure S20. Mass spectrum obtained of the open-chain form of 3,3,6,6-tetramethyl-9-phenyl-3,4,5,6,7,9-hexahydro-1*H*-xanthene-1,8(2*H*)-dione synthesized using the continuous flow reactor. This was sampled from the tenth 3 mL of reaction mixture to flow through this column.

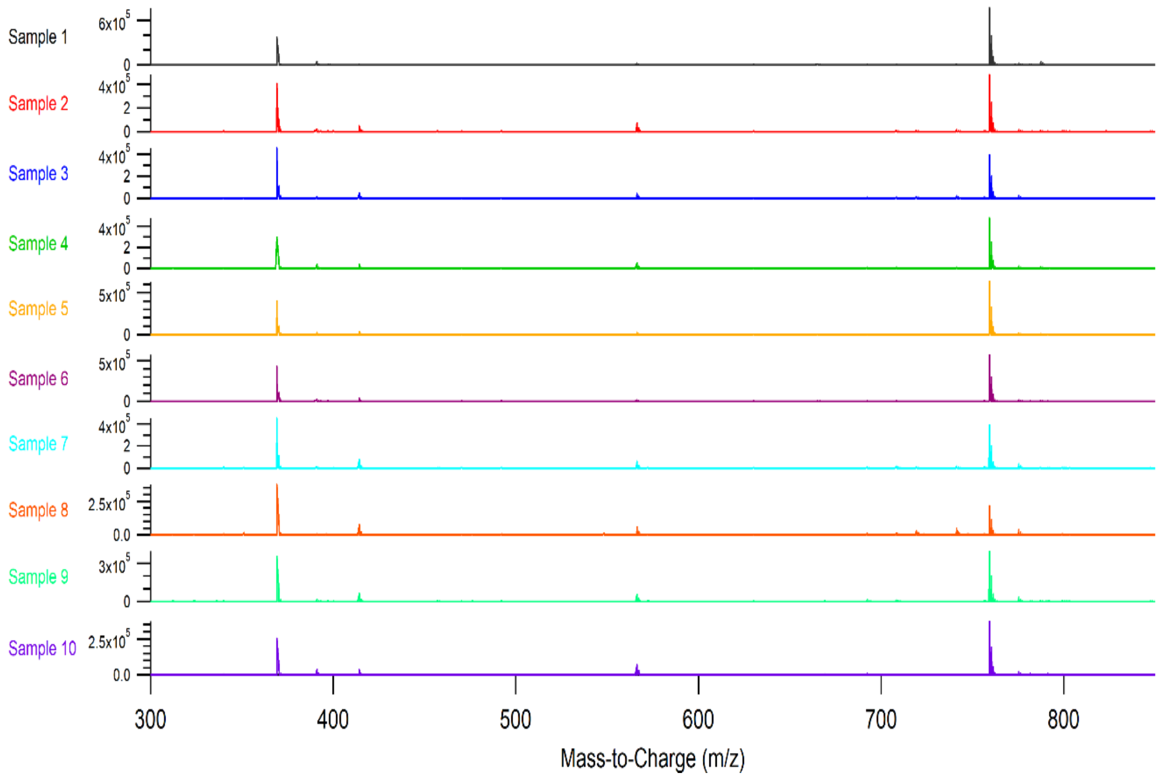

Figure S21. All of the mass spectra obtained of the open-chain form of 3,3,6,6-tetramethyl-9-phenyl-3,4,5,6,7,9-hexahydro-1*H*-xanthene-1,8(2*H*)-dione synthesized using the continuous flow reactor using the same column for all samples. Between each 3 mL injection of reaction mixture, 25 mL of ethanol was used to rinse out the tubing and column on the reactor. Each "sample" is representative of the product collected from each injection after it had excess solvent removed and recrystallized. This includes data from Figures S22 – S31.

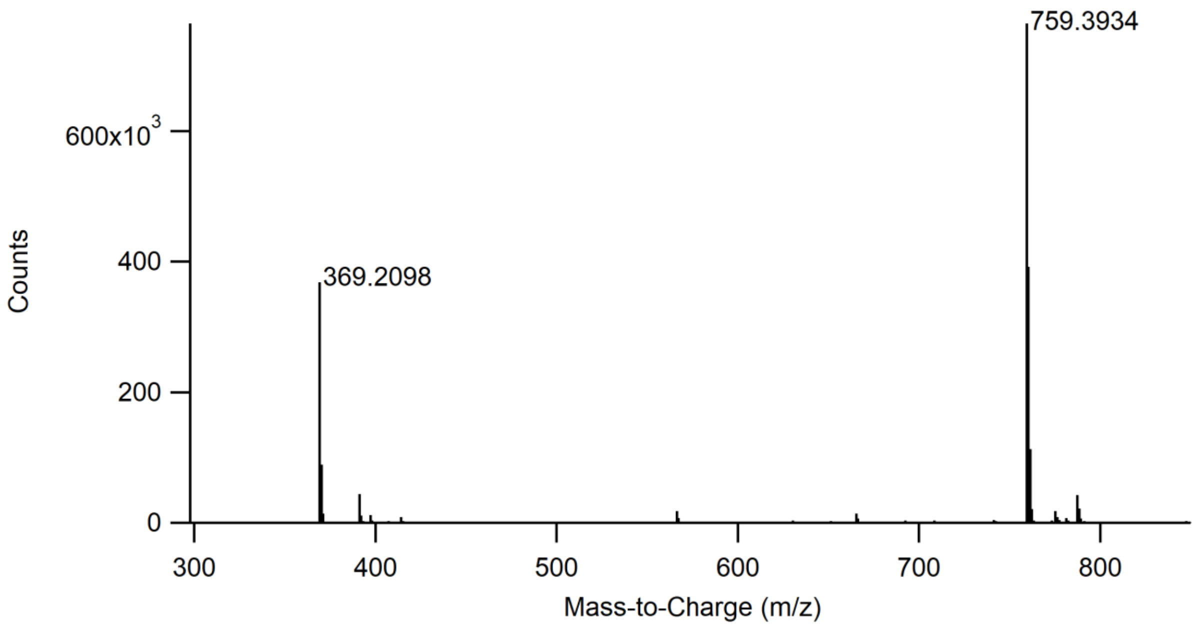

Figure S22. Mass spectrum obtained of the open-chain form of 3,3,6,6-tetramethyl-9-phenyl-3,4,5,6,7,9-hexahydro-1*H*-xanthene-1,8(2*H*)-dione synthesized using the continuous flow reactor. This was sampled from the first 3 mL of reaction mixture to flow through this column.

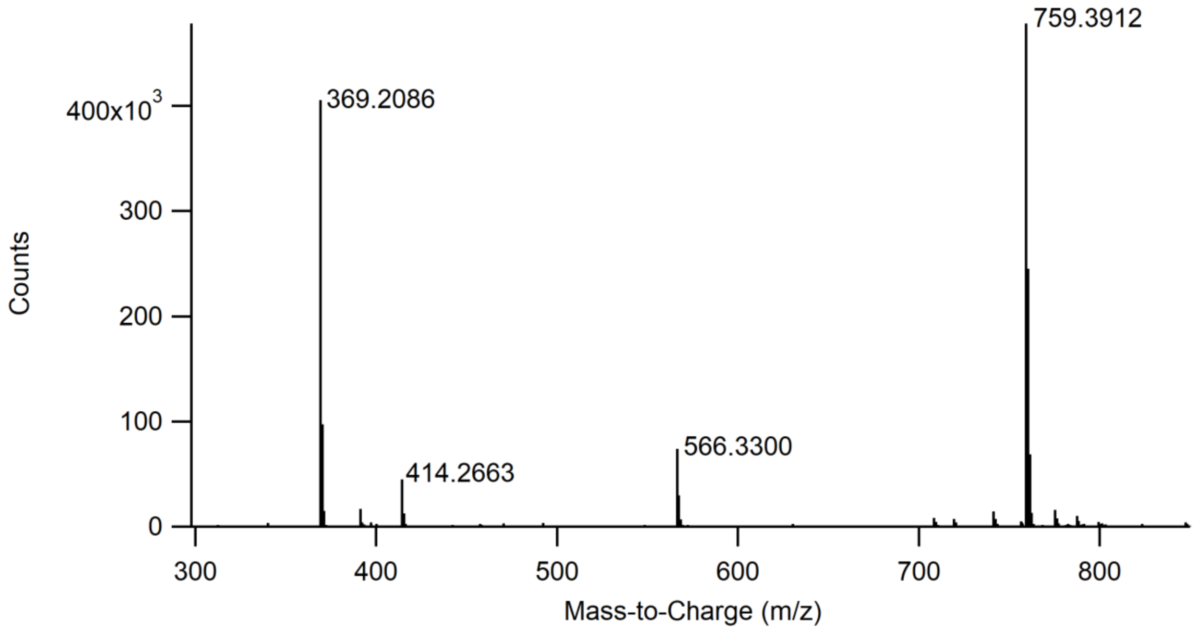

Figure S23. Mass spectrum obtained of the open-chain form of 3,3,6,6-tetramethyl-9-phenyl-3,4,5,6,7,9-hexahydro-1*H*-xanthene-1,8(2*H*)-dione synthesized using the continuous flow reactor. This was sampled from the second 3 mL of reaction mixture to flow through this column.

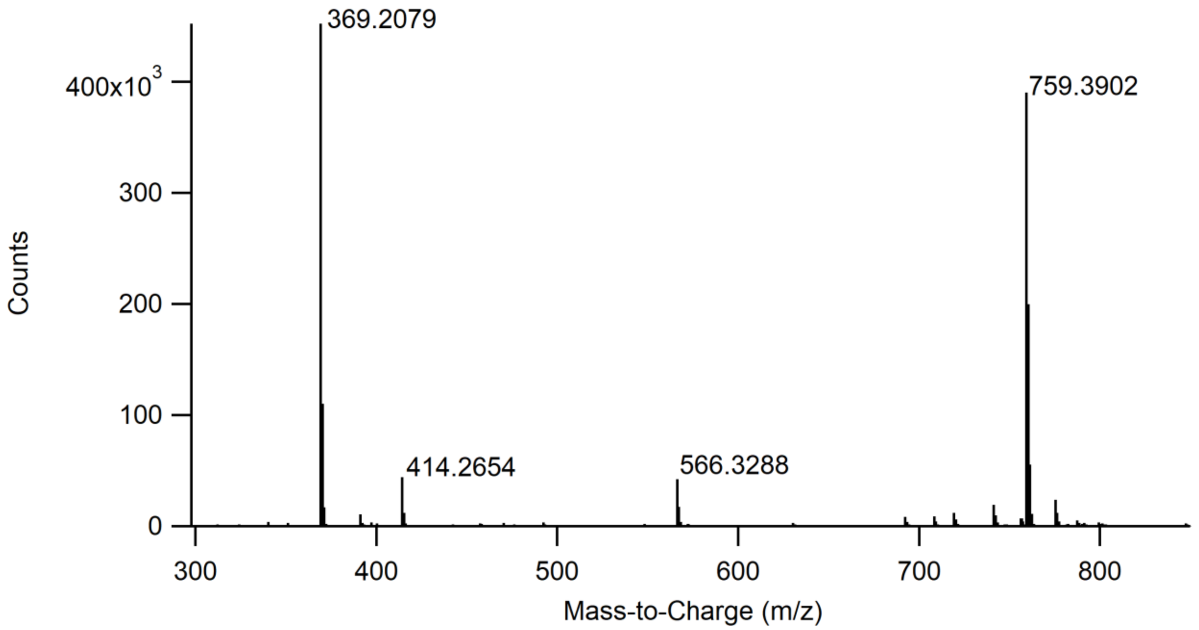

Figure S24. Mass spectrum obtained of the open-chain form of 3,3,6,6-tetramethyl-9-phenyl-3,4,5,6,7,9-hexahydro-1*H*-xanthene-1,8(2*H*)-dione synthesized using the continuous flow reactor. This was sampled from the third 3 mL of reaction mixture to flow through this column.

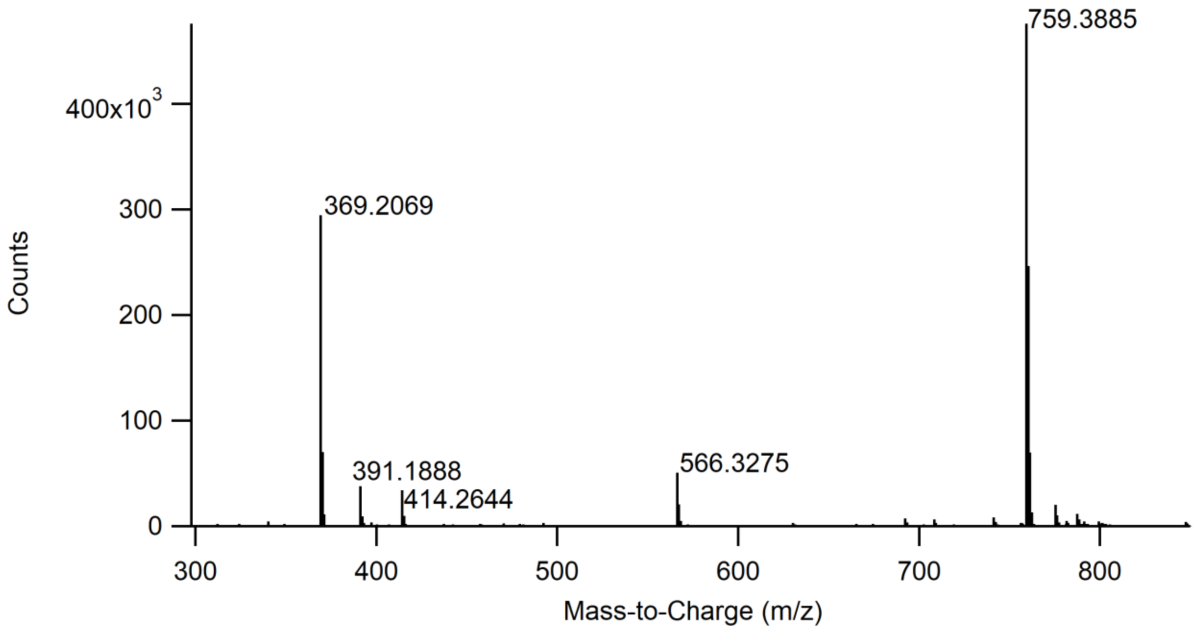

Figure S25. Mass spectrum obtained of the open-chain form of 3,3,6,6-tetramethyl-9-phenyl-3,4,5,6,7,9-hexahydro-1*H*-xanthene-1,8(2*H*)-dione synthesized using the continuous flow reactor. This was sampled from the fourth 3 mL of reaction mixture to flow through this column.

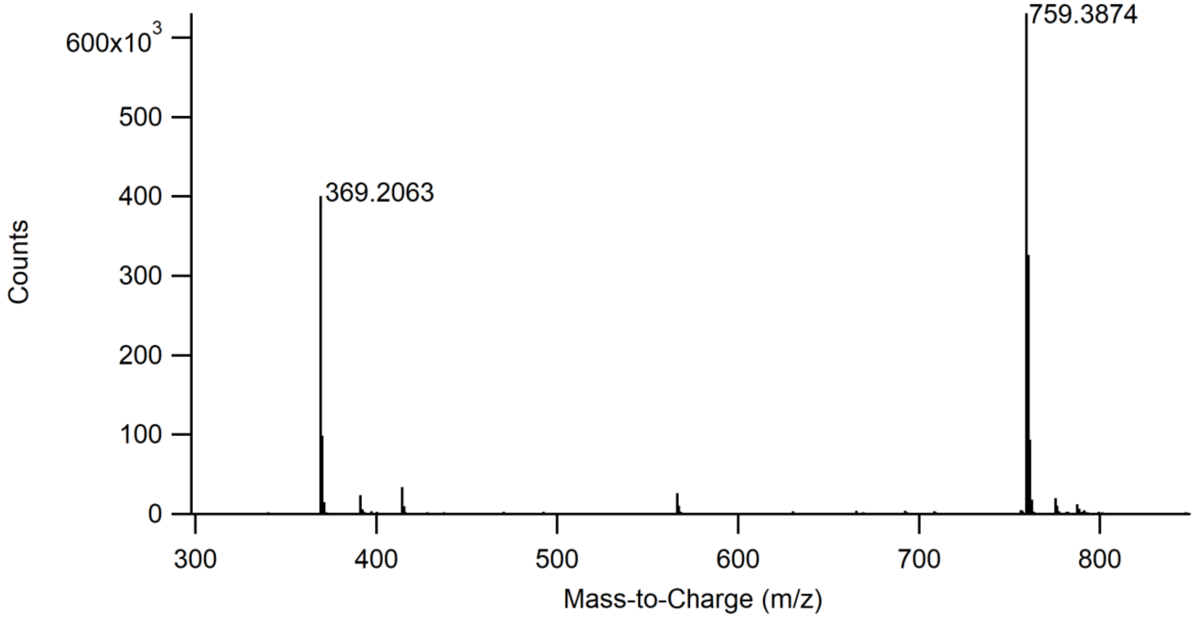

Figure S26. Mass spectrum obtained of the open-chain form of 3,3,6,6-tetramethyl-9-phenyl-3,4,5,6,7,9-hexahydro-1*H*-xanthene-1,8(2*H*)-dione synthesized using the continuous flow reactor. This was sampled from the fifth 3 mL of reaction mixture to flow through this column.

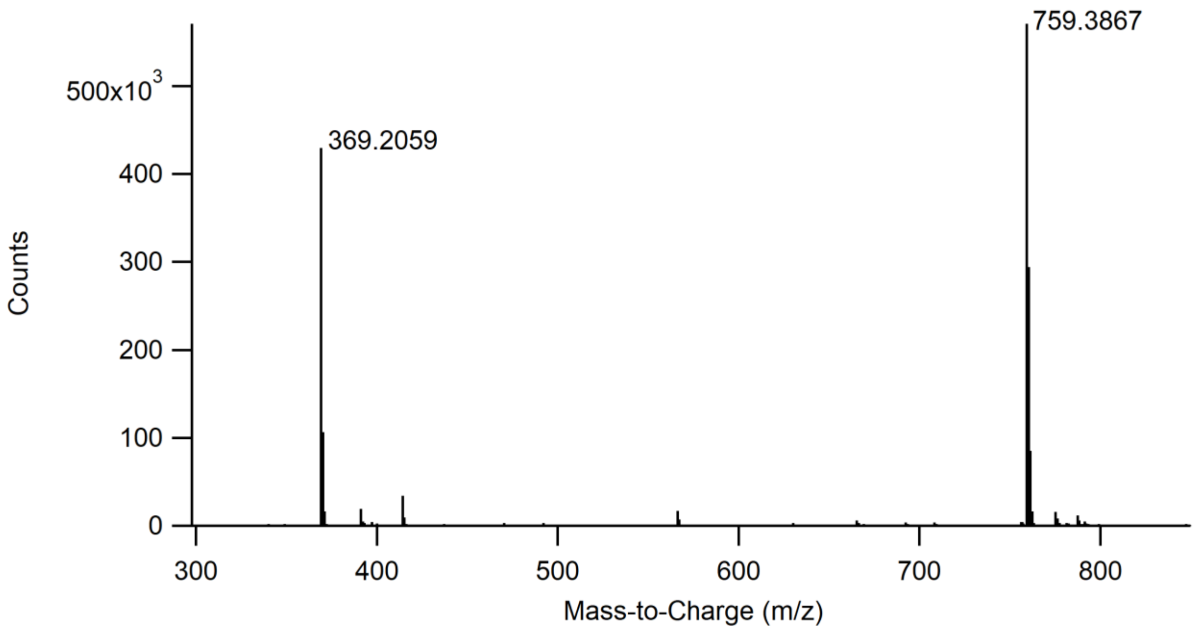

Figure S27. Mass spectrum obtained of the open-chain form of 3,3,6,6-tetramethyl-9-phenyl-3,4,5,6,7,9-hexahydro-1*H*-xanthene-1,8(2*H*)-dione synthesized using the continuous flow reactor. This was sampled from the sixth 3 mL of reaction mixture to flow through this column.

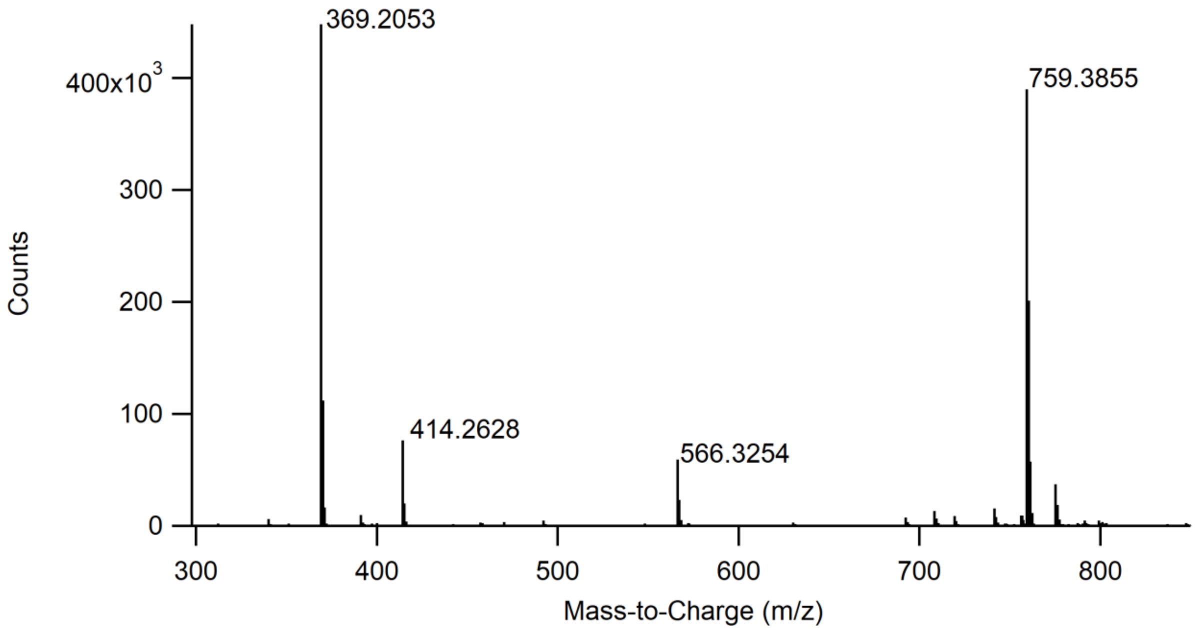

Figure S28. Mass spectrum obtained of the open-chain form of 3,3,6,6-tetramethyl-9-phenyl-3,4,5,6,7,9-hexahydro-1*H*-xanthene-1,8(2*H*)-dione synthesized using the continuous flow reactor. This was sampled from the seventh 3 mL of reaction mixture to flow through this column.

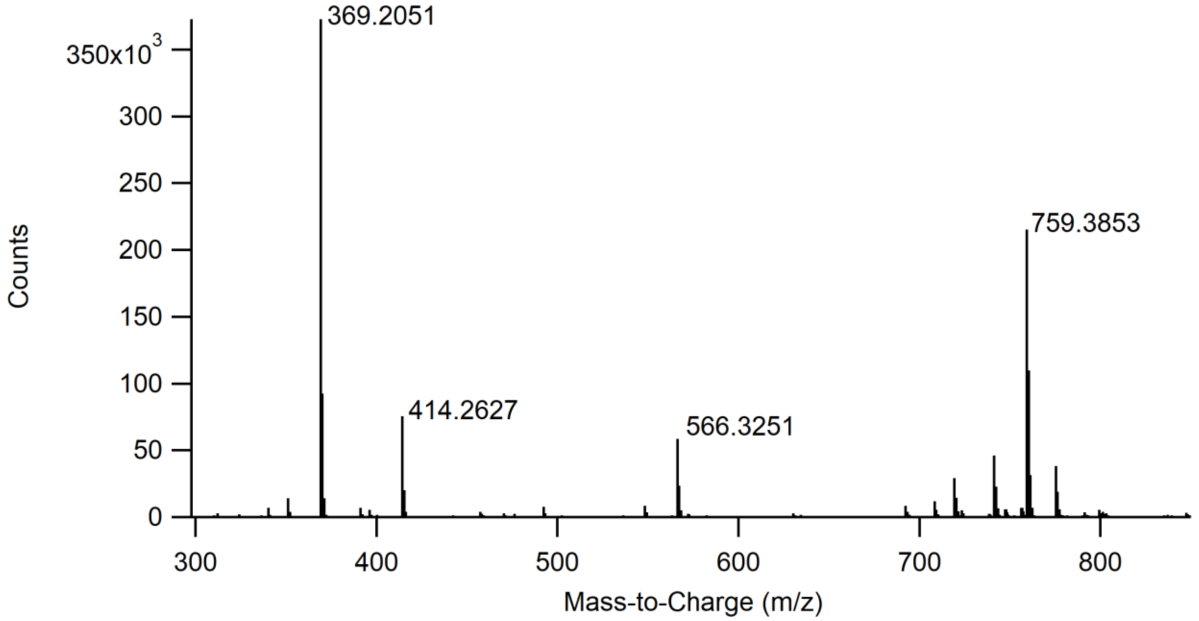

Figure S29. Mass spectrum obtained of the open-chain form of 3,3,6,6-tetramethyl-9-phenyl-3,4,5,6,7,9-hexahydro-1*H*-xanthene-1,8(2*H*)-dione synthesized using the continuous flow reactor. This was sampled from the eighth 3 mL of reaction mixture to flow through this column.

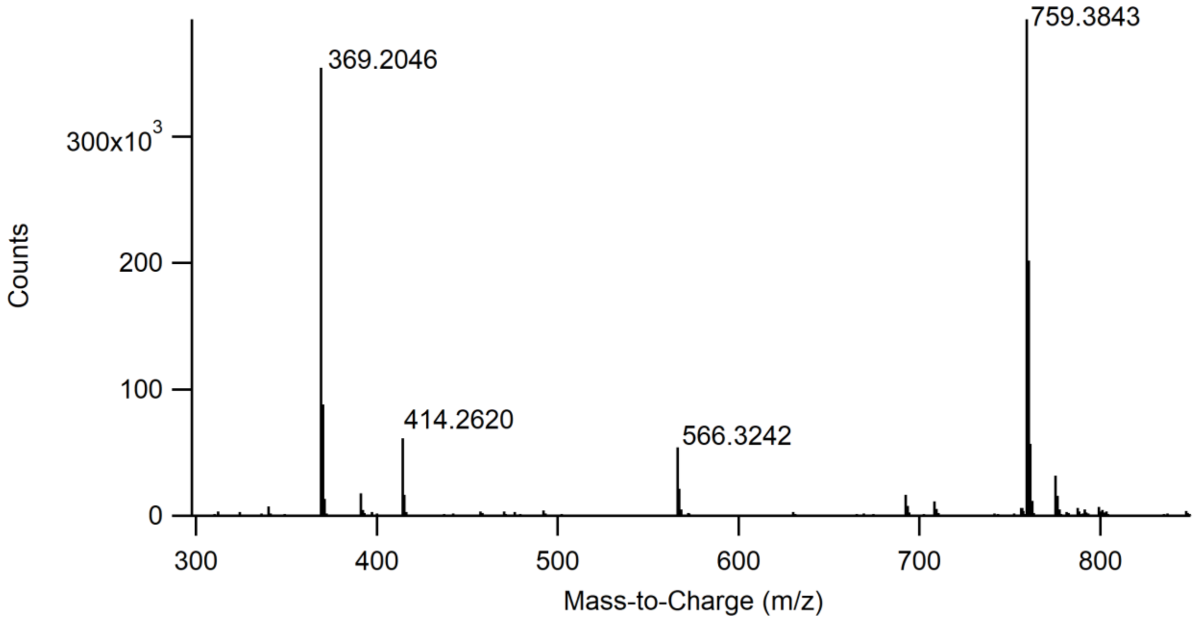

Figure S30. Mass spectrum obtained of the open-chain form of 3,3,6,6-tetramethyl-9-phenyl-3,4,5,6,7,9-hexahydro-1*H*-xanthene-1,8(2*H*)-dione synthesized using the continuous flow reactor. This was sampled from the ninth 3 mL of reaction mixture to flow through this column.

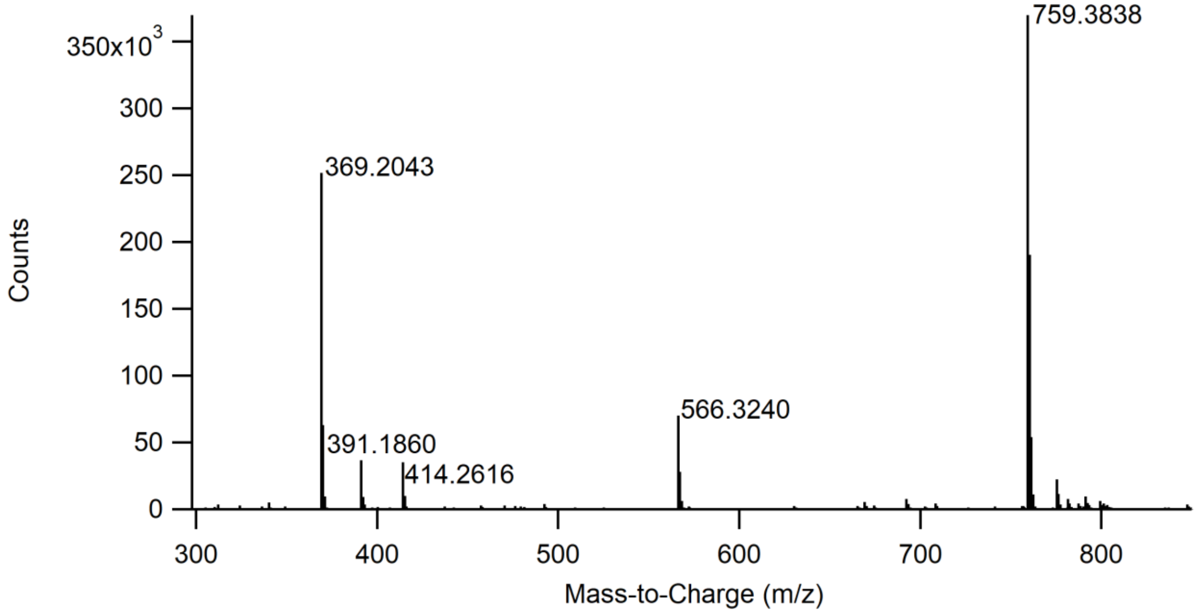

Figure S31. Mass spectrum obtained of the open-chain form of 3,3,6,6-tetramethyl-9-phenyl-3,4,5,6,7,9-hexahydro-1*H*-xanthene-1,8(2*H*)-dione synthesized using the continuous flow reactor. This was sampled from the tenth 3 mL of reaction mixture to flow through this column.

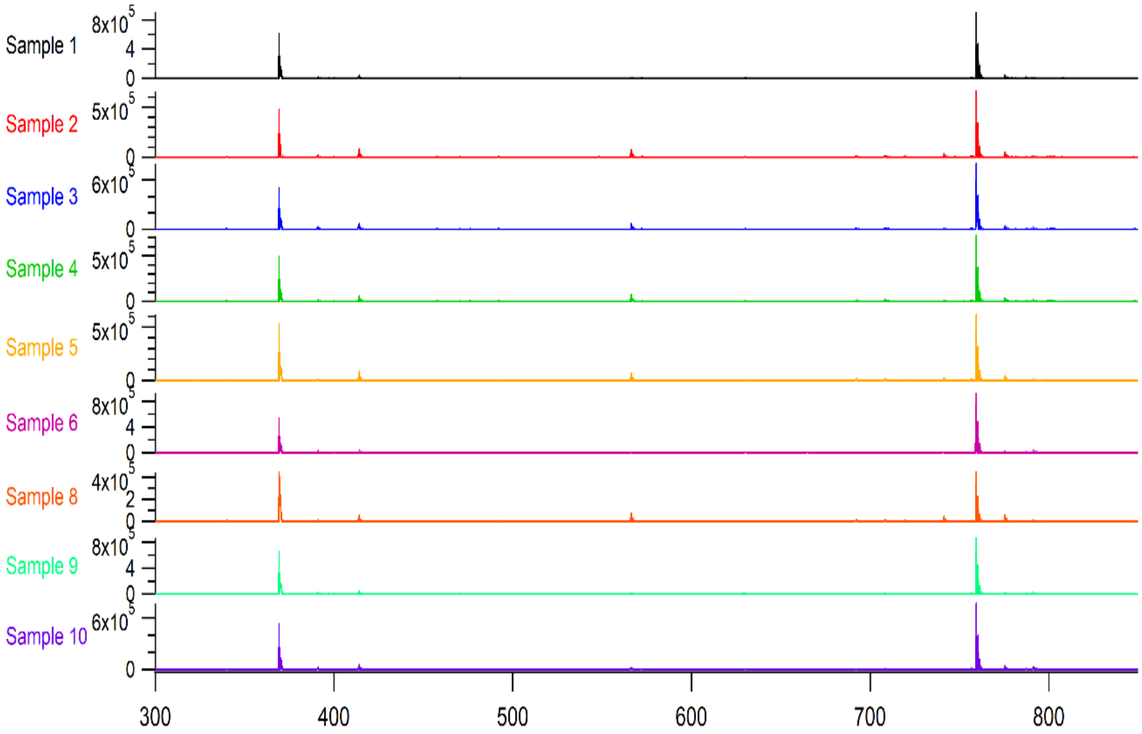

Figure S32. All of the mass spectra obtained of the open-chain form of 3,3,6,6-tetramethyl-9-phenyl-3,4,5,6,7,9-hexahydro-1*H*-xanthene-1,8(2*H*)-dione synthesized using the continuous flow reactor using the same column for all samples. Between each 3 mL injection of reaction mixture, 25 mL of ethanol was used to rinse out the tubing and column on the reactor. Each "sample" is representative of the product collected from each injection after it had excess solvent removed and recrystallized. This includes data from Figures S33 – S41. Note, in the experiments involving this set and column, an error occurred when Sample 7 was being collected causing the continuous flow system to continually pump over 20 mL of reaction mixture through the system without a rinse cycle. Because of this, data from Sample 7 was not included here. After the system was stopped, the system was rinsed with 25 mL of ethanol before proceeding with Sample 8. This provides further qualitative evidence that the column could be used indefinitely as there were no significant changes to be noted in the Samples collected after this error.

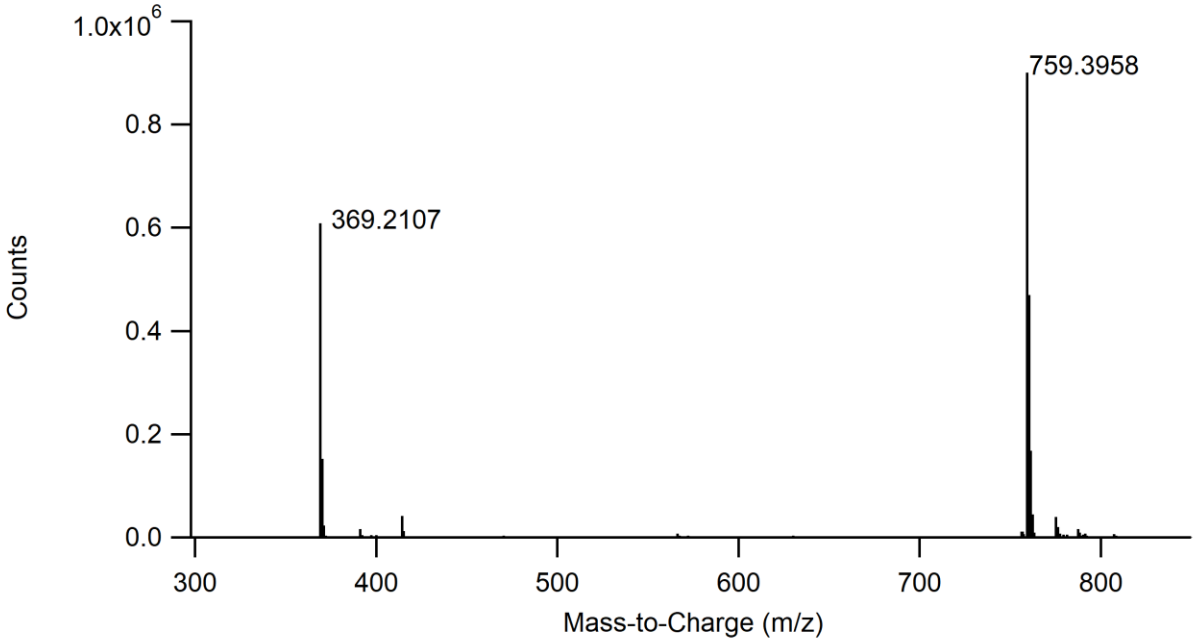

Figure S33. Mass spectrum obtained of the open-chain form of 3,3,6,6-tetramethyl-9-phenyl-3,4,5,6,7,9-hexahydro-1*H*-xanthene-1,8(2*H*)-dione synthesized using the continuous flow reactor. This was sampled from the first 3 mL of reaction mixture to flow through this column.

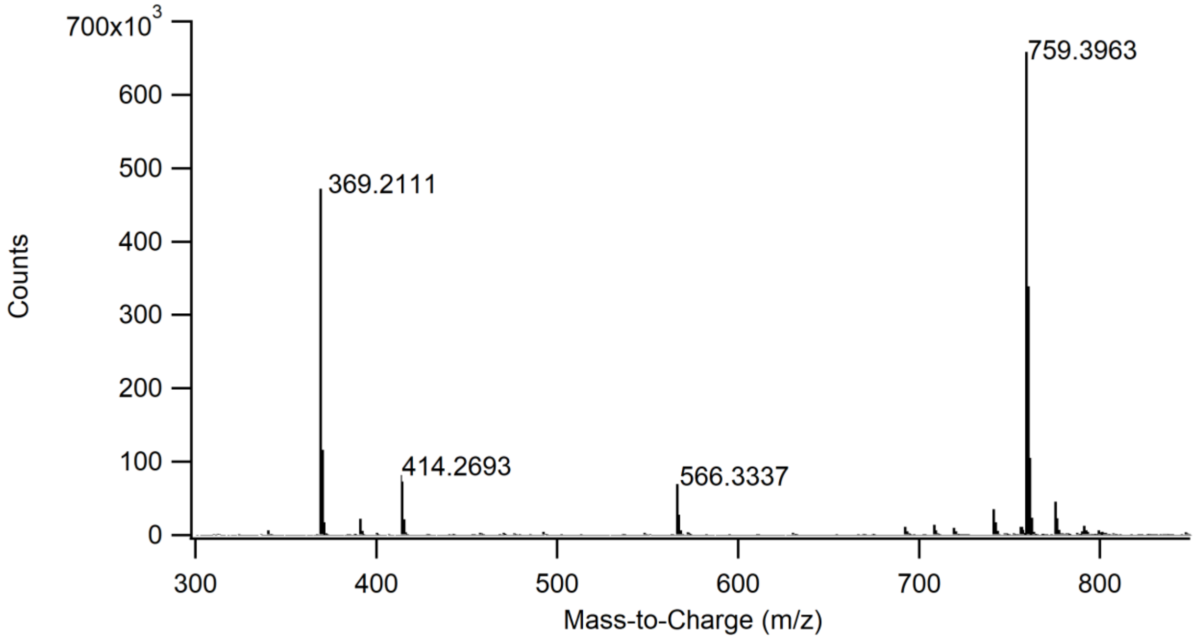

Figure S34. Mass spectrum obtained of the open-chain form of 3,3,6,6-tetramethyl-9-phenyl-3,4,5,6,7,9-hexahydro-1*H*-xanthene-1,8(2*H*)-dione synthesized using the continuous flow reactor. This was sampled from the second 3 mL of reaction mixture to flow through this column.

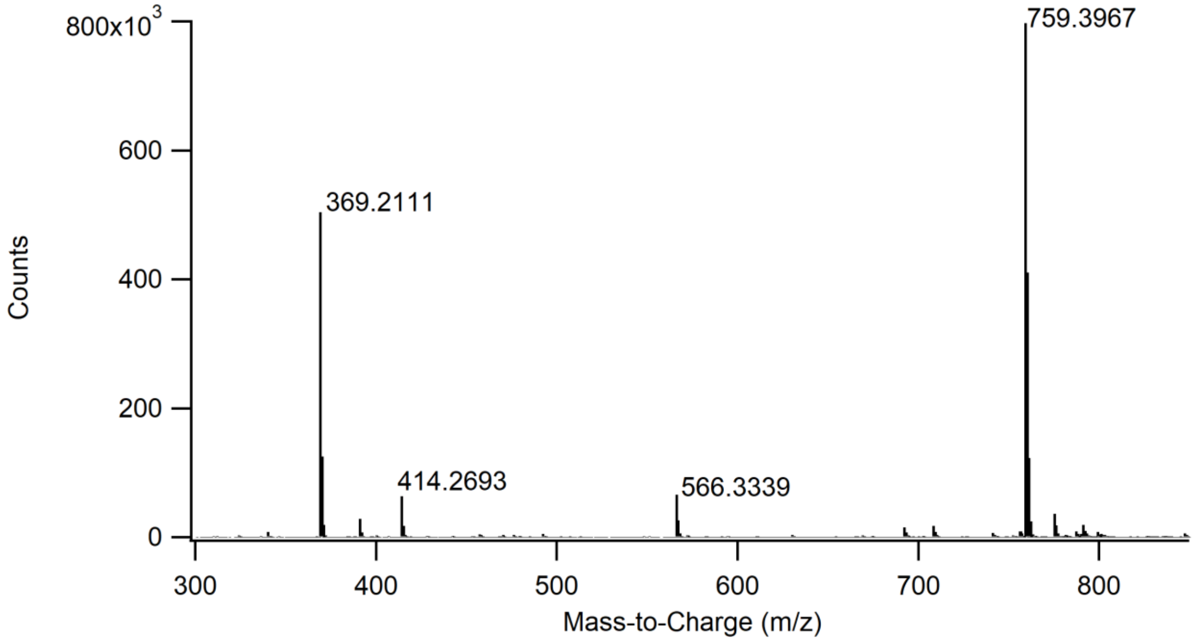

Figure S35. Mass spectrum obtained of the open-chain form of 3,3,6,6-tetramethyl-9-phenyl-3,4,5,6,7,9-hexahydro-1*H*-xanthene-1,8(2*H*)-dione synthesized using the continuous flow reactor. This was sampled from the third 3 mL of reaction mixture to flow through this column.

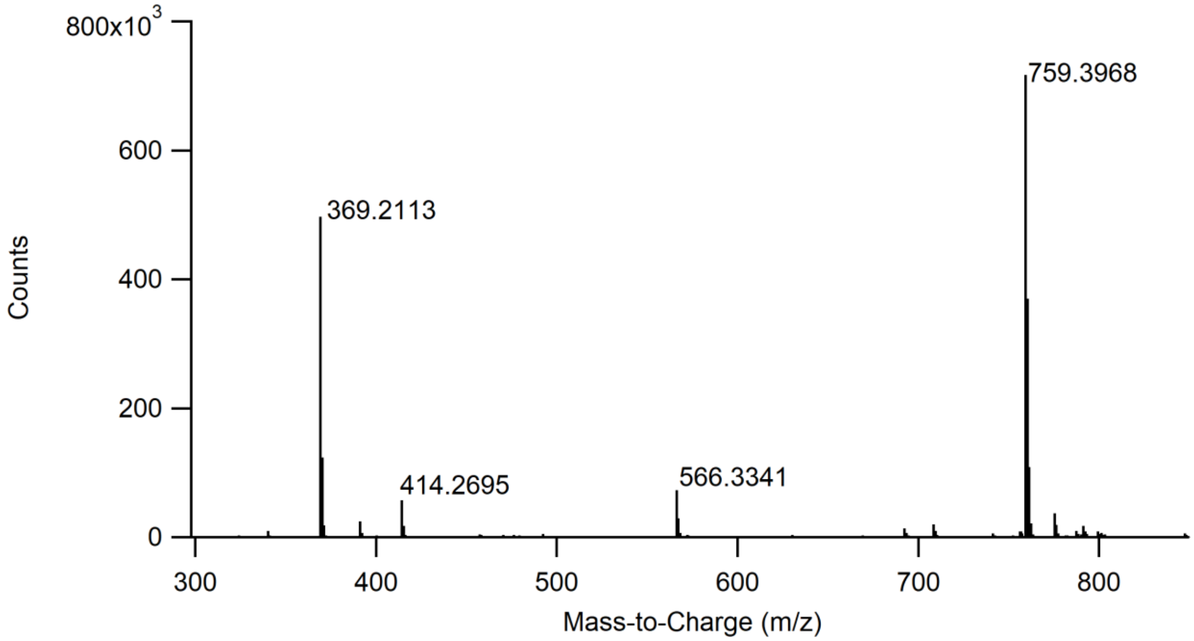

Figure S36. Mass spectrum obtained of the open-chain form of 3,3,6,6-tetramethyl-9-phenyl-3,4,5,6,7,9-hexahydro-1*H*-xanthene-1,8(2*H*)-dione synthesized using the continuous flow reactor. This was sampled from the fourth 3 mL of reaction mixture to flow through this column.

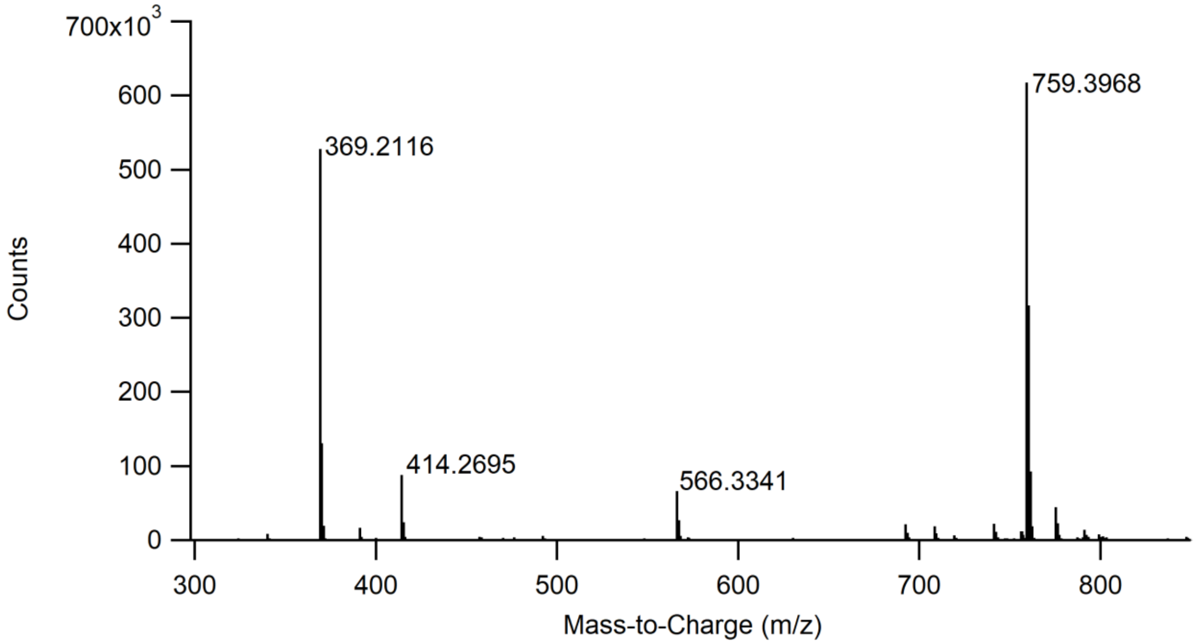

Figure S37. Mass spectrum obtained of the open-chain form of 3,3,6,6-tetramethyl-9-phenyl-3,4,5,6,7,9-hexahydro-1*H*-xanthene-1,8(2*H*)-dione synthesized using the continuous flow reactor. This was sampled from the fifth 3 mL of reaction mixture to flow through this column.

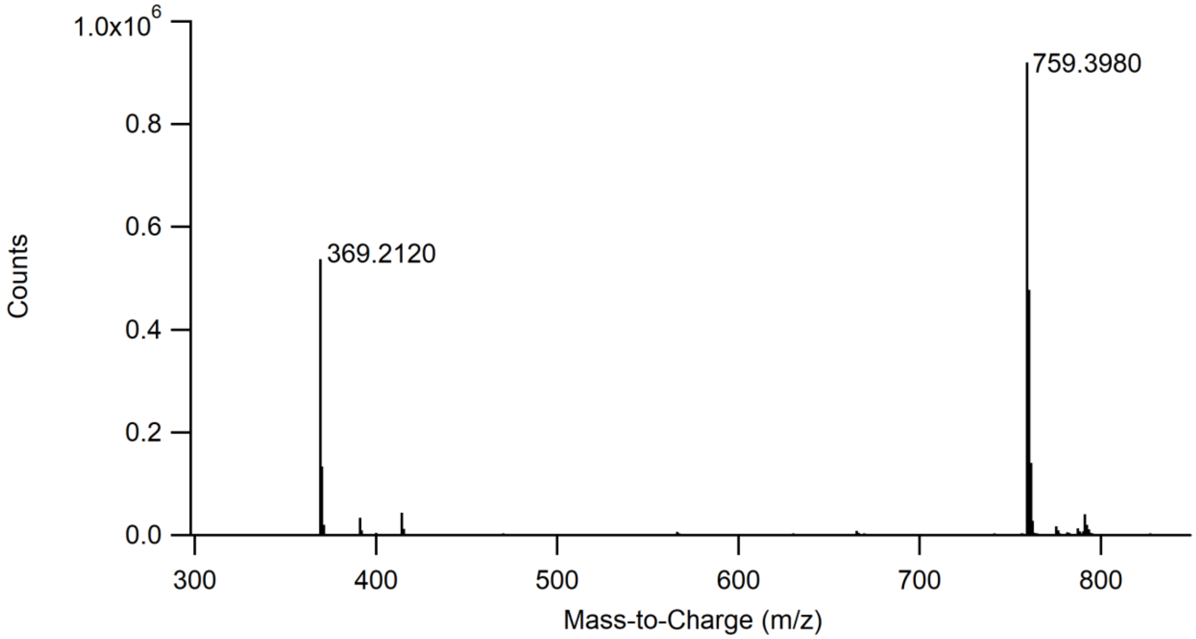

Figure S38. Mass spectrum obtained of the open-chain form of 3,3,6,6-tetramethyl-9-phenyl-3,4,5,6,7,9-hexahydro-1*H*-xanthene-1,8(2*H*)-dione synthesized using the continuous flow reactor. This was sampled from the sixth 3 mL of reaction mixture to flow through this column.

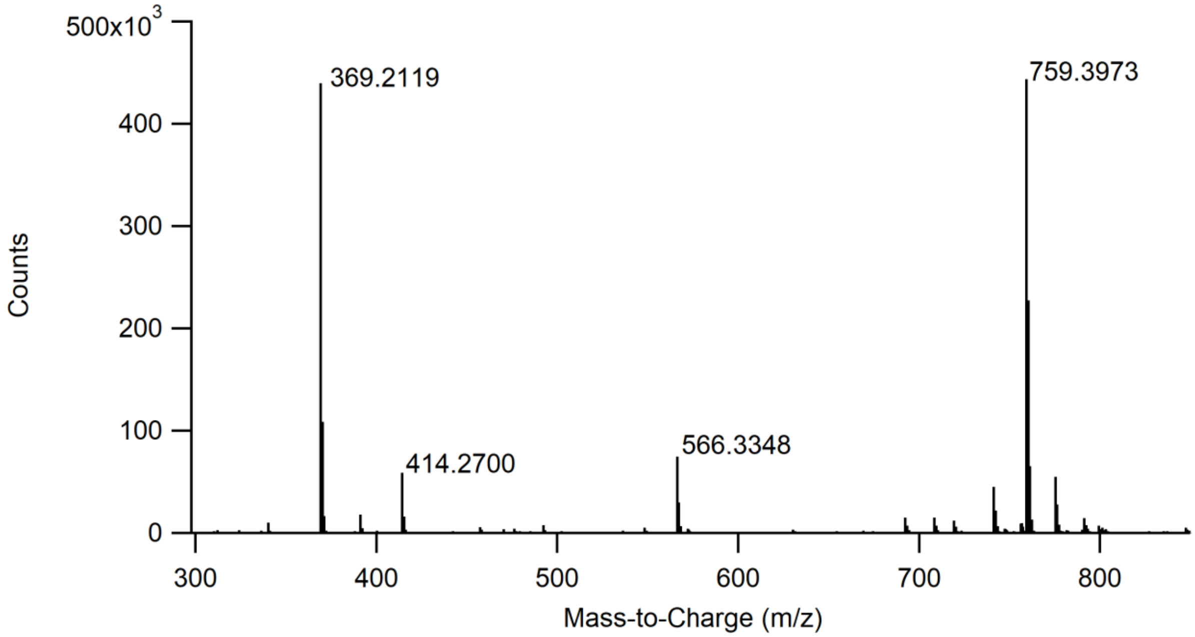

Figure S39. Mass spectrum obtained of the open-chain form of 3,3,6,6-tetramethyl-9-phenyl-3,4,5,6,7,9-hexahydro-1*H*-xanthene-1,8(2*H*)-dione synthesized using the continuous flow reactor. This was sampled from the "eighth" 3 mL of reaction mixture to flow through this column.

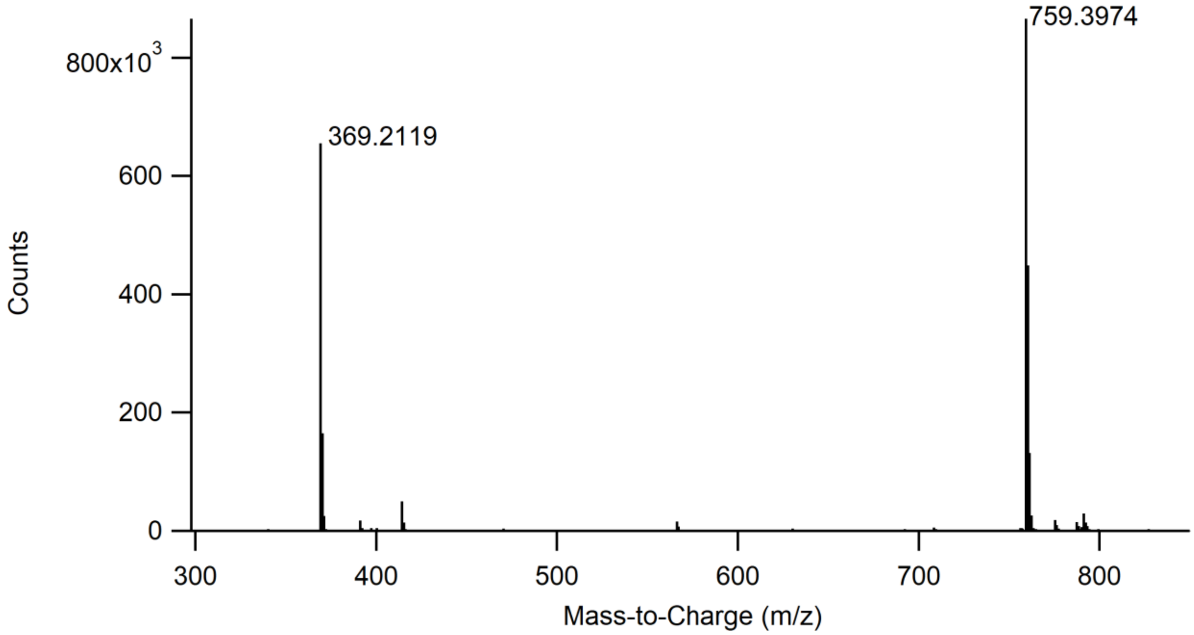

Figure S40. Mass spectrum obtained of the open-chain form of 3,3,6,6-tetramethyl-9-phenyl-3,4,5,6,7,9-hexahydro-1*H*-xanthene-1,8(2*H*)-dione synthesized using the continuous flow reactor. This was sampled from the "ninth" 3 mL of reaction mixture to flow through this column.

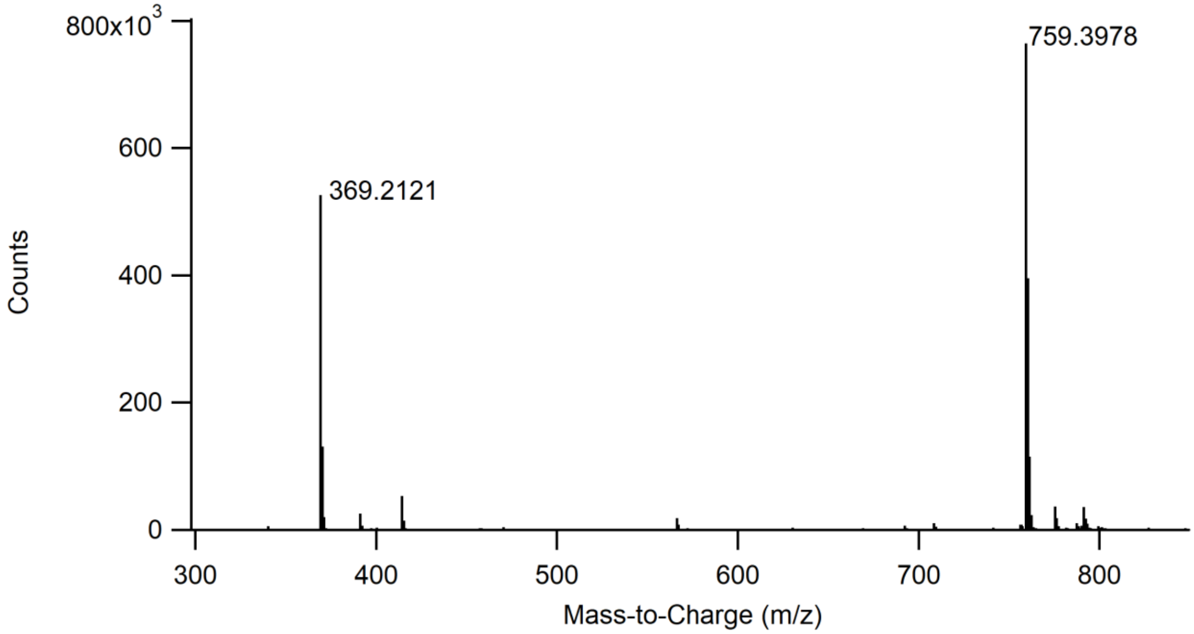

Figure S41. Mass spectrum obtained of the open-chain form of 3,3,6,6-tetramethyl-9-phenyl-3,4,5,6,7,9-hexahydro-1*H*-xanthene-1,8(2*H*)-dione synthesized using the continuous flow reactor. This was sampled from the "tenth" 3 mL of reaction mixture to flow through this column.

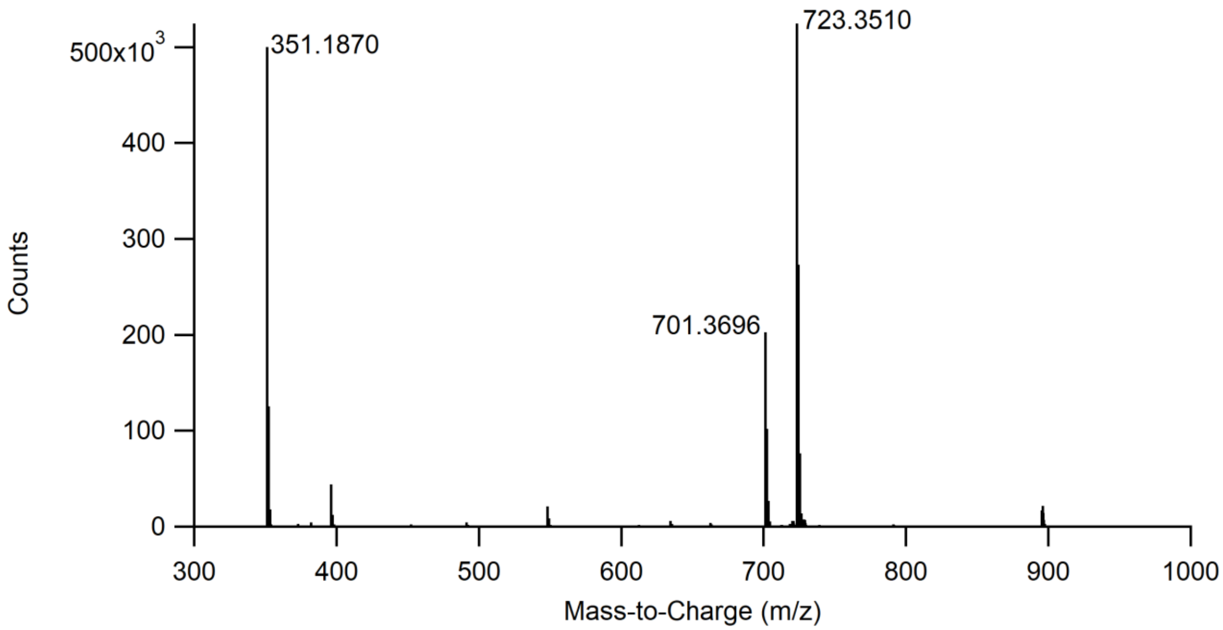

Figure S42. Mass spectrum of a purchased standard of 3,3,6,6-tetramethyl-9-phenyl-3,4,5,6,7,9-hexahydro-1*H*-xanthene-1,8(2*H*)-dione.

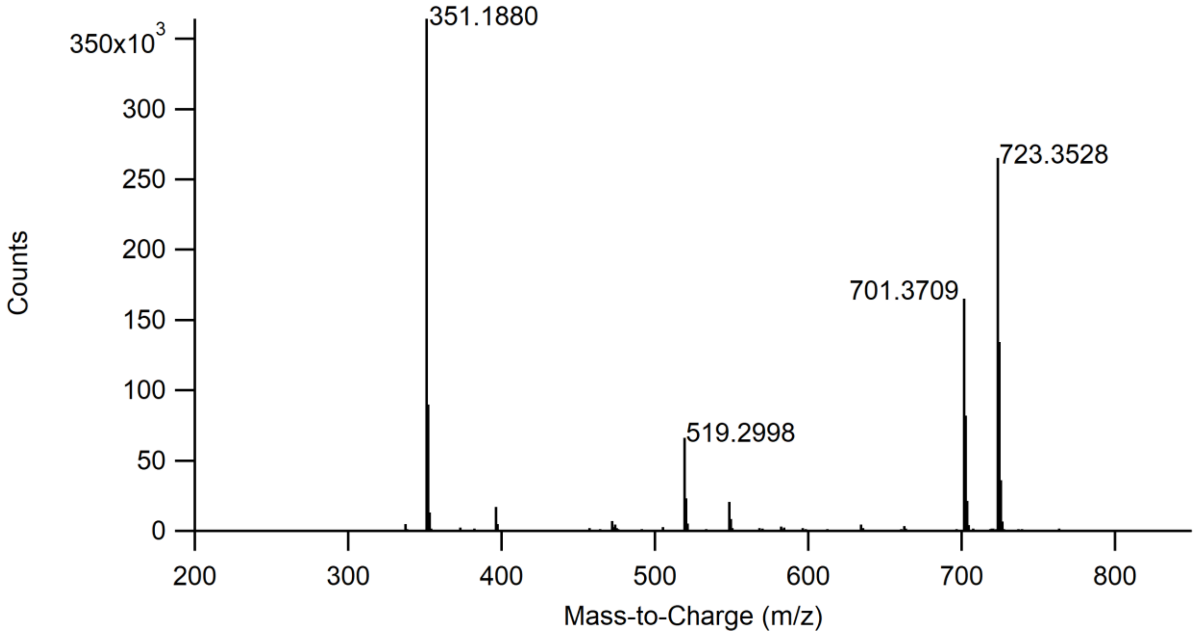

Figure S43. Mass spectrum of 3,3,6,6-tetramethyl-9-phenyl-3,4,5,6,7,9-hexahydro-1*H*-xanthene-1,8(2*H*)-dione synthesized from the continuous flow reactor after an H^+ work up.

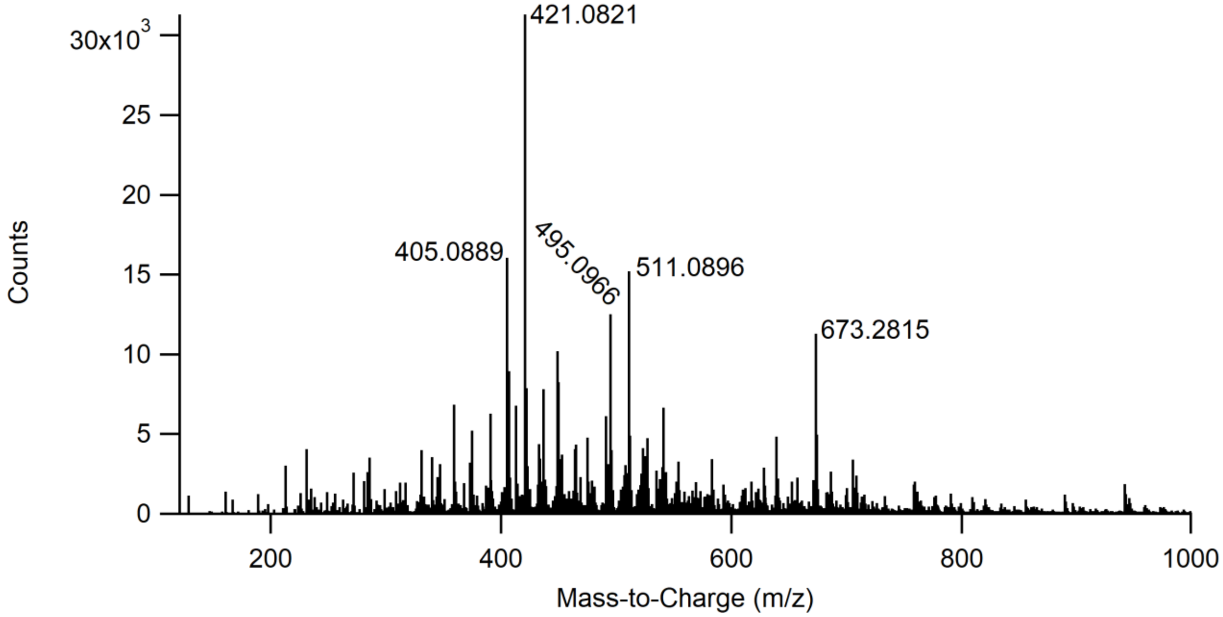

Figure S44. Mass spectrum of benzaldehyde used as a starting material for the reactions performed in this manuscript.

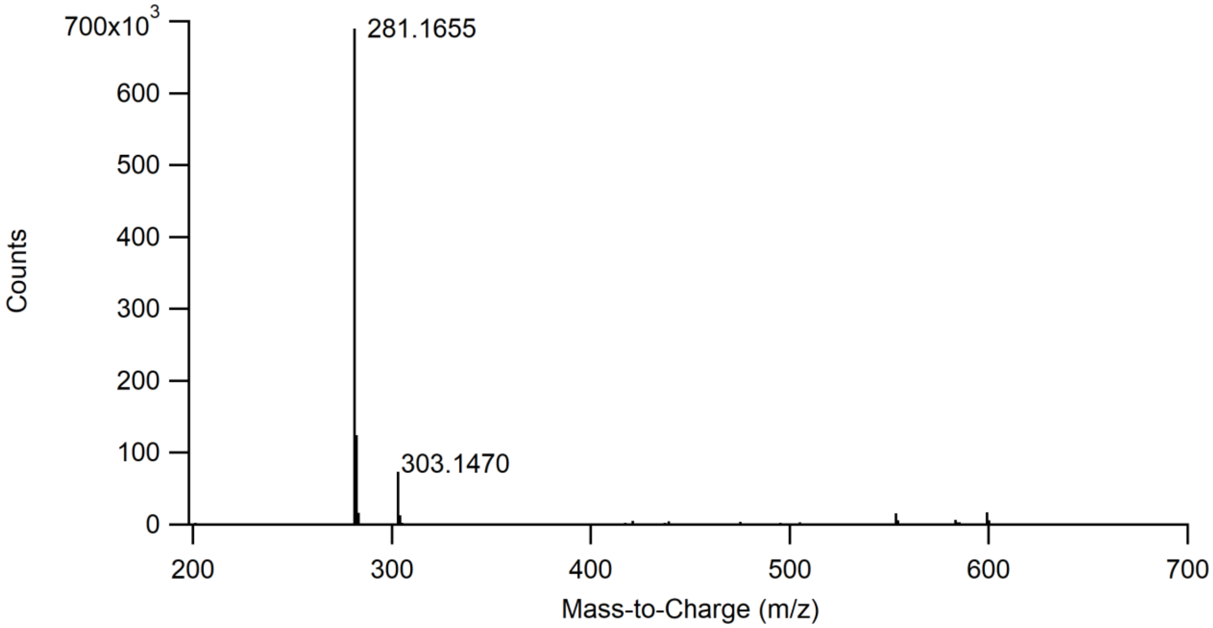

Figure S45. Mass spectrum of the dimedone used as a starting material for the reactions performed in this manuscript.

Table S2. Elemental analysis (via ICP-HRMS) performed by Hazen Research Inc. to detect copper in product samples.

**Sample**	**Amount of Copper Detected**
Eluent from Column	130 µg/L
Open Chain Product Sample 1	4.6 µg/g
Open Chain Product Sample 2	2.0 µg/g
Open Chain Product Sample 3	2.2 µg/g
Ethanol Blank	<10 µg/L

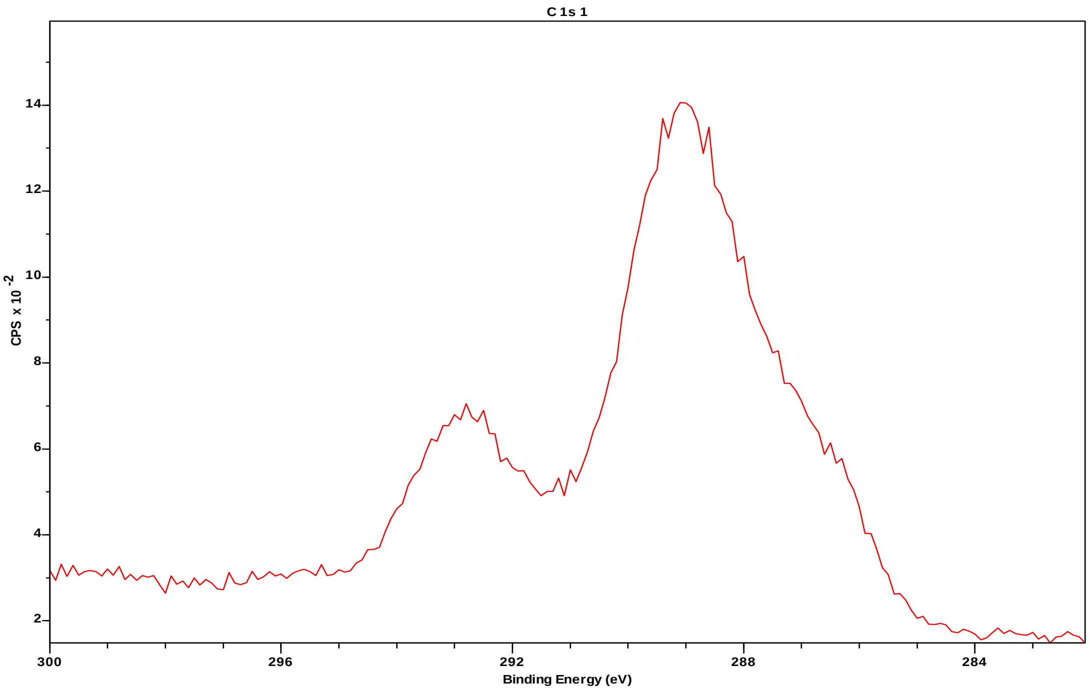

Figure S46. High-resolution scan of Carbon 1s region of the CuBTC MOF using XPS before it was used for catalysis.

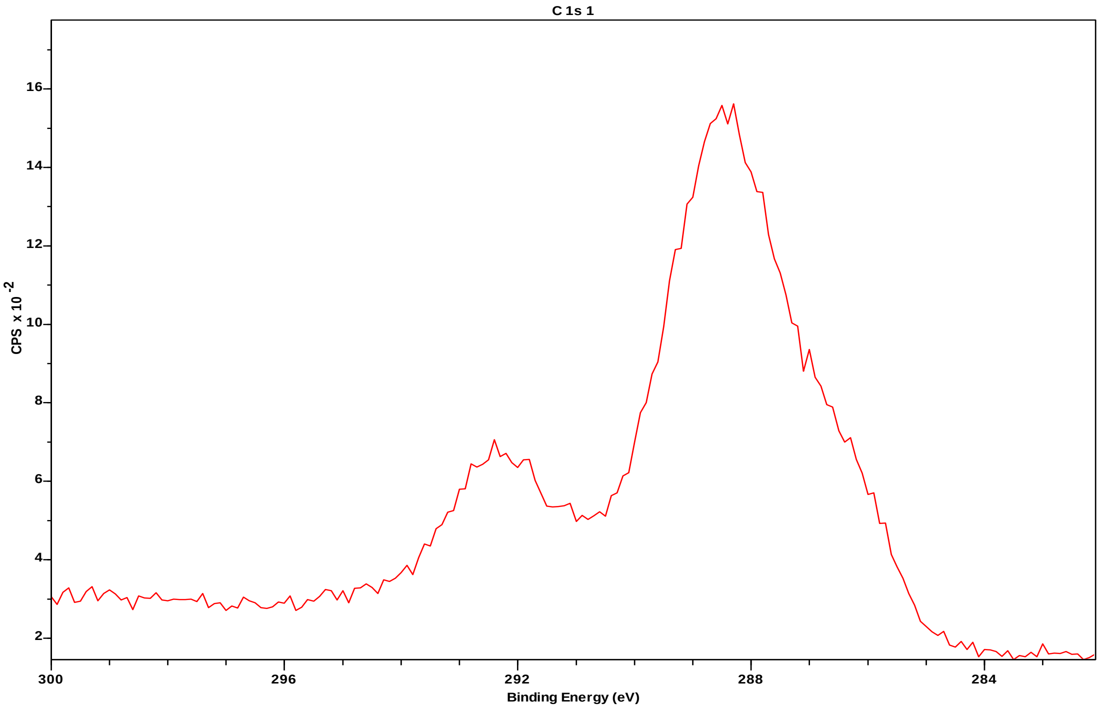

Figure S47. High-resolution scan of Carbon 1s region of the CuBTC MOF using XPS after it was used for catalysis under continuous flow conditions.

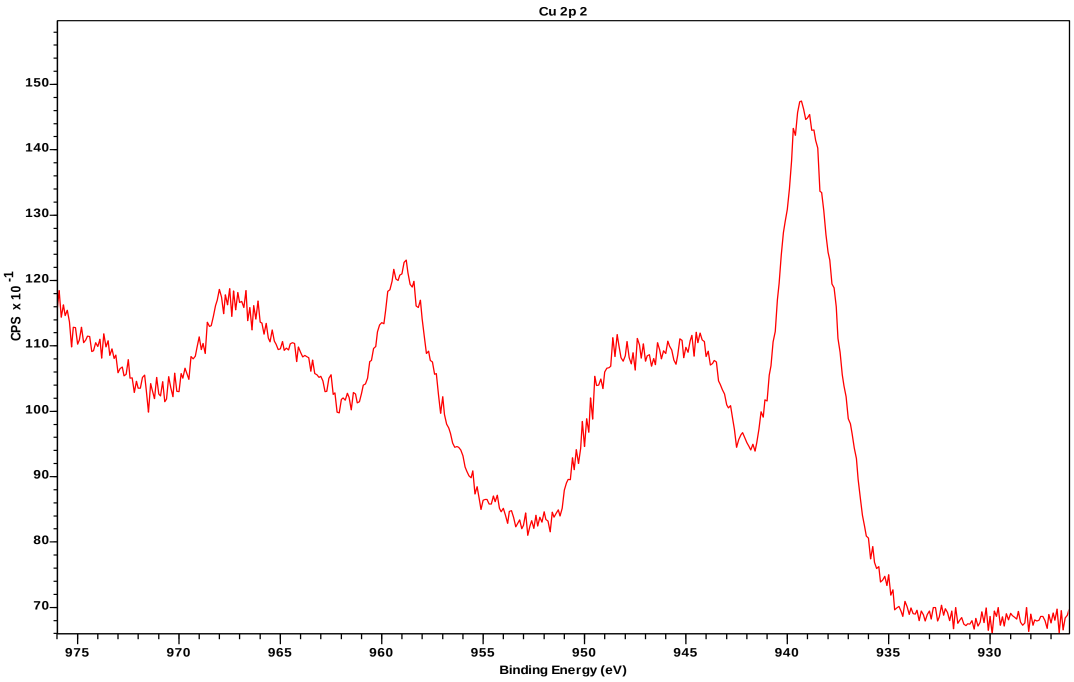

Figure S48. High-resolution scan of Copper 2p region of the CuBTC MOF using XPS before it was used for catalysis.

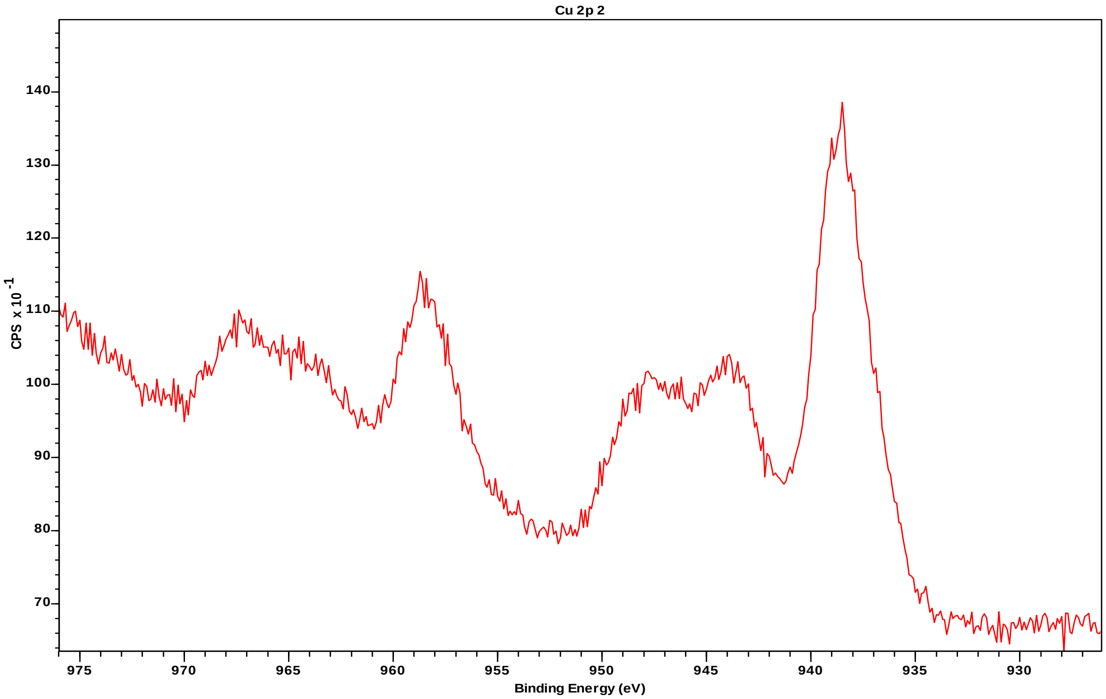

Figure S49. High-resolution scan of Copper 2p region of the CuBTC MOF using XPS after it was used for catalysis under continuous flow conditions.

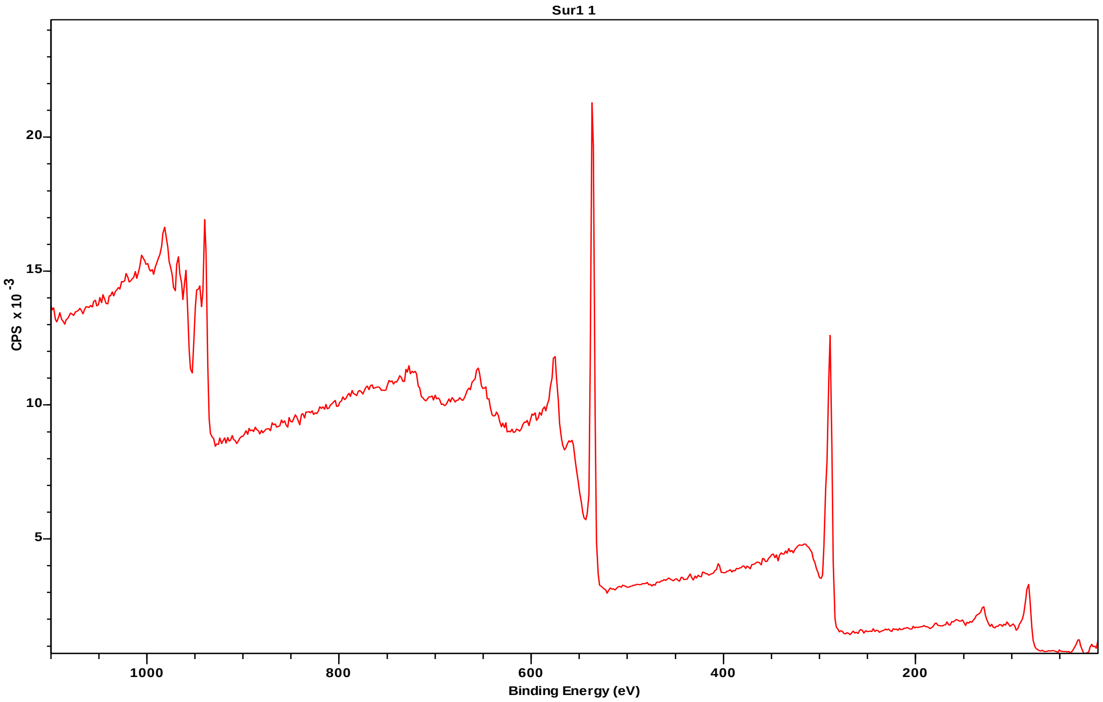

Figure S50. Surface scan of the CuBTC MOF using XPS before it was used for catalysis under continuous flow conditions.

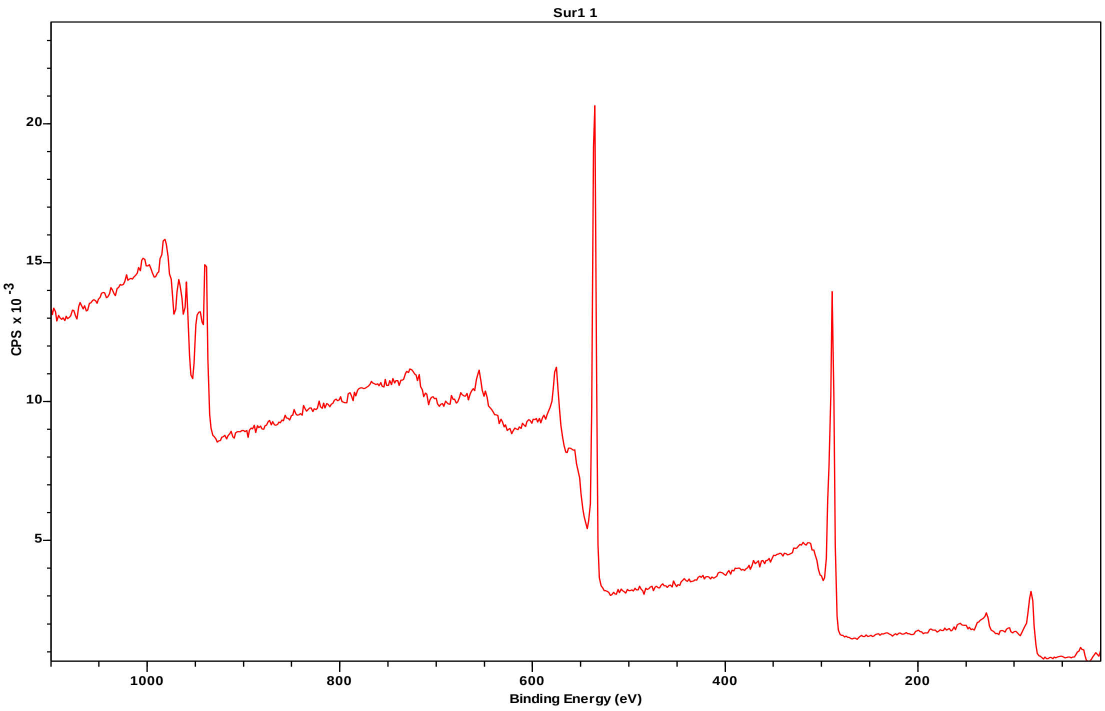

Figure S51. Surface scan of the CuBTC MOF using XPS after it was used for catalysis under continuous flow conditions.
